# Milk-derived extracellular vesicles alleviate ulcerative colitis by regulating the gut immunity and reshaping the gut microbiota

**DOI:** 10.7150/thno.62046

**Published:** 2021-07-25

**Authors:** Lingjun Tong, Haining Hao, Zhe Zhang, Youyou Lv, Xi Liang, Qiqi Liu, Tongjie Liu, Pimin Gong, Lanwei Zhang, Fangfang Cao, Giorgia Pastorin, Chuen Neng Lee, Xiaoyuan Chen, Jiong-Wei Wang, Huaxi Yi

**Affiliations:** 1 College of Food Science and Engineering, Ocean University of China, 5 Yushan Road, Qingdao 266003, P. R. China; 2 Department of Surgery, Yong Loo Lin School of Medicine, National University of Singapore, 1E Kent Ridge Road, Singapore 119228, Singapore; 3 Nanomedicine Translational Research Programme, Centre for NanoMedicine, Yong Loo Lin School of Medicine, National University of Singapore, Singapore 117609, Singapore; 4 Departments of Diagnostic Radiology, Yong Loo Lin School of Medicine, National University of Singapore, Singapore 119074, Singapore; 5 Department of Pharmacy, Faculty of Science, National University of Singapore, Singapore 117543, Singapore; 6 Department of Chemical and Biomolecular Engineering, and Department of Biomedical Engineering, Faculty of Engineering, National University of Singapore, Singapore 117575, Singapore; 7 Clinical Imaging Research Centre, Centre for Translational Medicine, Yong Loo Lin School of Medicine, National University of Singapore, Singapore 117599, Singapore; 8 Cardiovascular Research Institute (CVRI), National University Heart Centre Singapore (NUHCS), 14 Medical Drive, Singapore 117599, Singapore; 9 Department of Physiology, Yong Loo Lin School of Medicine, National University of Singapore, 2 Medical Drive, Singapore 117593, Singapore; 10 Key Laboratory of Precision Nutrition and Food Quality, Department of Nutrition and Health, China Agricultural University, Beijing 100083, China

**Keywords:** Extracellular vesicles, ulcerative colitis, Treg/Th17 cell balance, intestinal immunity, gut microbiome

## Abstract

**Rationale:** Bovine milk constitutes an essential part of human diet, especially for children, due to its enrichment of various nutrients. We recently developed an effective protocol for the isolation of extracellular vesicles from milk (mEVs) and discovered that mEVs contained large amounts of immune-active proteins and modulated the gut immunity and microbiota in healthy mice. Here, we aimed to explore the therapeutic effects of mEVs on inflammatory bowel disease.

**Methods:** MicroRNAs and protein content in mEVs were analyzed by RNA sequencing and proteomics, respectively, followed by functional annotation. Ulcerative colitis (UC) was induced by feeding mice with dextran sulfate sodium. Intestinal immune cell populations were phenotyped by flow cytometry, and the gut microbiota was analyzed *via* 16S rRNA sequencing.

**Results:** We showed that abundant proteins and microRNAs in mEVs were involved in the regulation of immune and inflammatory pathways and that oral administration of mEVs prevented colon shortening, reduced intestinal epithelium disruption, inhibited infiltration of inflammatory cells and tissue fibrosis in a mouse UC model. Mechanistically, mEVs attenuated inflammatory response *via* inhibiting TLR4-NF-κB signaling pathway and NLRP3 inflammasome activation. Furthermore, mEVs were able to correct cytokine production disorder and restore the balance between T helper type 17 (Th17) cells and interleukin-10^+^Foxp3^+^ regulatory T (Treg) cells in the inflamed colon. The disturbed gut microbiota in UC was also partially recovered upon treatment with mEVs. The correlation between the gut microbiota and cytokines suggests that mEVs may modulate intestinal immunity *via* influencing the gut microbiota.

**Conclusions:** These findings reveal that mEVs alleviate colitis by regulating intestinal immune homeostasis *via* inhibiting TLR4-NF-κB and NLRP3 signaling pathways, restoring Treg/Th17 cell balance, and reshaping the gut microbiota.

## Introduction

Ulcerative colitis (UC) is an idiopathic inflammatory bowel disease (IBD) that involves the colonic mucosal immune responses triggered by microbial antigens and presents recurrent symptoms such as diarrhea, abdominal pain and bloody stool 1, 2. The mucosa of UC patients contains a large number of macrophages and T cells, which secrete excess amount of pro-inflammatory mediators such as cytokines 3. While the pathogenesis of UC remains unknown, hyperactivation of Toll-like receptors (TLRs) and NOD-, LRR- and pyrin domain-containing protein 3 (NLRP3) inflammasome have been considered critical as suppression of TLR4 and NLRP3 signaling pathways could effectively inhibit the production of inflammatory cytokines and prevent the progression of UC 4, 5. Emerging evidence indicates that TLR4-myeloid differentiation factor 88-nuclear factor kappa-B (TLR4-Myd88-NF-κB) pathway regulates the balance between the interleukin-10 (IL-10)-producing regulatory T (Treg) cells and the interleukin-17A (IL-17A)-producing T helper type 17 (Th17) cells in UC 6. This Treg/Th17 cell balance in the intestine is also regulated by the gut microbiota 7 and disturbed by imbalanced intestinal flora 8. Therefore, targeting TLR4/NLRP3 signaling pathways and the Th17/Treg axis emerges as a new therapeutic strategy for the treatment of UC.

The TLR4 signaling pathway contributing to necrotizing enterocolitis, a devastating intestinal disease characterized by infection and inflammation in the colons of newborns, can be inhibited by breast milk 9. However, the exact components in the milk that exert intestine-protective effects remain elusive. Extracellular vesicles (EVs) are emerging as critical regulators in the resident immune cells and the gut microbiota, especially in the development of inflammatory diseases, including atherosclerosis, and UC 10-12. Bovine milk, as part of human diet, contains a large amount of EVs (mEVs), which originate from mammary epithelial cells and are released into the milk *via* the endosomal route or direct budding from the plasma membrane 13, 14. mEVs contain a large number of immune-related microRNAs and proteins, and do not induce any systemic toxicity or adverse immune response 15, 16. A recent study reported that porcine mEV microRNA protected the intestine from lipopolysaccharide (LPS)-induced injury *via* inhibiting NF-κB and p53 pathways 10. miRNAs in mEVs have been shown to influence the development of the immune system by targeting Treg cells 17.

mEVs regulate intercellular signaling, inflammation and immune response, thus protect against stress and various disease conditions 18. Although the field of mEVs has witnessed rapid progress over the past few years, the underlying mechanisms by which mEVs alleviate autoimmune and inflammatory diseases remain to be explored. Our previous study indicates that mEVs enhance intestinal immunity in normal mice 19. In this study, we investigated the effects of mEVs on intestinal immune homeostasis and gut microbiota in a mouse model of UC.

## Methods

### Preparation and characterization of mEVs

Raw milk from Holstein cows during the mid-lactation period was obtained from a local dairy farm. To avoid bacterial contamination and the production of bacteria-derived vesicles, the standard method of milking was adopted. The samples were placed in a portable freezer and returned to the laboratory within 1 h. 0.05% chymosin and 0.3% CaCl_2_ were added to the raw milk and incubated at 4 °C for 30 min. The sample was centrifuged at 16,500 × g at 4 °C for 30 min to remove fat globules, casein aggregates and residual chymosin. The supernatant was then centrifuged at 100,000× g using HIMAC CP70ME Ultracentrifuge (Hitachi, Ltd., Tokyo, Japan) at 4 °C for 60 min to remove large particles and residual debris. The supernatant was subjected to ultracentrifugation at 135,000× g at 4 °C for 90 min. Following resuspension with phosphate-buffered saline (PBS), the pelleted EVs were washed by ultracentrifugation at 135,000× g (60 min). mEVs were transferred onto the 100 kDa filters and centrifuged at 5,000× g for 30 min twice, and then resuspended in PBS. The protein concentration of mEVs was determined using a Bicinchoninic Acid (BCA) kit (Beyotime Biotechnology, CN). mEVs were stored at 4 °C before use. The complete methodology for EV isolation was submitted to EV-TRACK (ID: EV200018).

The morphology of mEVs was visualized using transmission electron microscopy (TEM). 10 μL mEVs were loaded on a copper grid covered by the formvar film, which was incubated for 1 min on ice. After negative staining with 2% phosphotungstic acid, mEVs was imaged under a HITACHI H-7650 Transmission Electron Microscope.

The size distribution was analyzed by ZetaView PMX 110 (Particle Metrix, Meerbusch, Germany). Briefly, mEVs were diluted in PBS and analyzed at 23~37 °C. All samples were analyzed in 4 replicates.

### Proteome analysis and miRNA sequencing of mEVs

The proteins of mEVs were separated on SDS-PAGE gel and digested by filter-aided sample preparation (FASP) as described previously 20. The sample was analyzed by Q Exactive (Thermo Scientific, MA, USA) coupled to Easy-nLC for 120 min. The peptide mixture was loaded onto a nanoViper Acclaim PepMap100 C18 column (100 μm × 2 cm) and separated on a C18-reversed phase analytical column (10 cm long, 75 μm inner diameter, 3 μm resin) in mobile phases A (0.1% Formic acid) and B (84% acetonitrile and 0.1% Formic acid) with a linear gradient of 0-55% B (110 min), 55-100% B (5 min), and held in 100% B (5 min) at a flow rate of 300 nL/min. Gene Ontology (GO) and Kyoto Encyclopedia of Genes and Genomes (KEGG) annotation of exosomal proteins were analyzed using Blast2GO (Version 3.3.5, https://www.blast2go.com/) and KEGG database (http://geneontology.org/).

Total RNA was extracted from 500 μg mEVs using RNeasy Plus Micro Kit (74034, QIAGEN) according to the manufacturer's instructions. The quality of extracted RNA was assessed using Agilent 2100 Bioanalyzer (Agilent Technologies, Santa Clara, CA, USA). The RNA libraries were constructed and subjected to purity and quality check using Agilent 2100 DNA Bioanalyzer and Quant-iT PicoGreen dsDNA assay kit. Quantitated libraries were analyzed by single end sequencing on Illumina Genome Analyzer (Shanghai Personal Biotechnology Co., Ltd, Shanghai, China). The miRNA target annotation and KEGG were analyzed using miRanda database (http://www.miranda.org/).

### In vitro assay

RAW264.7 cells were obtained from the Cell Bank of the Chinese Academy of Sciences (Shanghai, China), and cultured in high-glucose DMEM medium supplemented with 10% fetal bovine serum (FBS), 100 U/mL penicillin, and 100 μg/mL streptomycin at 37 ℃ in a 5% CO_2_ atmosphere. RAW264.7 cells were seeded and incubated in 6/48-well plates (1× 10^5^ cells/well) for 12 h, and then treated with different concentrations of mEVs for 8 h. Then, LPS (100 ng/mL, from *Escherichia coli* O55:B5, Sigma-Aldrich Co., Shanghai, CN) was added and incubated for 20 h. The nitrite accumulation in the supernatant was measured using nitric oxide assay kit (Nanjingjiancheng Bio, Jiangsu, CN). For cytotoxicity assay, RAW264.7 cells were plated overnight at a density of 4 × 10^4^ cells/well in 96-well plates, followed by 24 h incubation with mEVs and LPS (100 ng/mL). 50 μL MTT solution (1 mg/mL) was added into wells and incubated at 37 °C for 4 h. 150 µL dimethyl sulfoxide (DMSO) was added to dissolve the purple formazan crystals, and the absorbance was measured at 570 nm with Multiskan FC.

To quantify the cellular uptake of mEV *in vitro*, the resuspended mEVs were labeled with PKH26 using PKH26 Red Fluorescent Cell Linker Mini Kit (Sigma-Aldrich, Shanghai, CN) as previously described 16. In brief, 500 μL resuspended mEVs were incubated with 4 μL PKH26 for 5 min in the dark, and 2 mL of 0.5% BSA-PBS was added to bind the excess dye for 10 min. The residual free dyes were removed by 100,000× g ultracentrifugation for 1 h. Finally, mEVs were transferred onto the 100 kDa filters and centrifuged twice, then resuspended in PBS. PKH26 with an equal concentration was added to PBS and processed as described above. RAW264.7 cells were cultured in DMEM supplemented with 10% EVs-depleted FBS (FBS was diluted and ultracentrifuged at 100,000× g for 120 min to eliminate bovine serum EVs 21) and plated onto confocal dish (Nest Biotechnology, Wuxi, CN). Following incubation with PKH26-labeled mEVs, cells were washed twice with PBS, fixed with 4% paraformaldehyde, and permeabilized in Triton-X 100 (0.1% in PBS) for 3 min. After permeabilization, samples were washed twice with PBS, and stained with 100 nM FITC Phalloidin in 1% BSA-PBS for 30 min in the dark. The cell nucleus was labeled with DAPI. Cellular uptake of mEVs was observed using a laser scanning confocal microscope (Fluo viewTM FV1000, Olympus, Tokyo, Japan).

### In vivo assay

Specific-pathogen-free (SPF) male C57BL/6 mice (7-8 weeks old) were purchased from the Laboratory Animal Breeding Center of Pengyue (Jinan, China). Mice were housed at 22 °C for 12 h light/dark cycles and maintained in individual cages. The standard diet (Table S5) and deionized water were provided *ad libitum*. UC was induced in mice by adding 3% (wt/vol) dextran sulfate sodium (DSS, Yeasen Biotech, Shanghai, CN). Mice were divided into five groups, including a control group designed as previously reported 12, DSS group (3% DSS), DSS + mEVs-L group (3% DSS + 0.6 mg/kg/day, low-dose), DSS + mEVs-M group (3% DSS + 1.8 mg/kg/day, medium-dose) and DSS + mEVs-H group (3% DSS + 3.0 mg/kg/day, high-dose). Mice received predetermined doses of mEVs *via* oral gavage every day. The development of colitis was monitored daily by assessing body weight and the presence of blood in the stool.

The distribution of mEVs *in vivo* was determined with fluorescently labeled mEVs in mice. mEVs were labeled by incubation with 15 μM 1,1-dioctadecyl-3,3,3,3-tetramethylindotricarbocyanine iodide (DiR, AAT Bioquest, Sunnyvale, CA, USA) at 37 ℃ for 30 min. DiR-EVs were centrifuged at 100,000× g for 1 h to remove residual dyes and other impurities such as lipoproteins. The control was prepared by incubation of DiR in PBS, and processed as described above for mEVs. DiR-EVs (0.5 mg) were administered to C57BL/6 mice by oral gavage or tail vein injection. The distribution of DiR-EVs *in vivo* was visualized using a PerkinElmer IVIS Lumina XRMS Ⅲ (Waltham, MA).

All experiments were performed according to the National Institutes of Health guide for the care and use of laboratory animals, and approved by the Committee on the ethics of animal experiments of the Ocean University of China (The permission number: spxy20190715215).

### Flow cytometric analysis

Mouse lamina propria mononuclear cells (LPMCs) were obtained from the colon as described previously 7. Cells were treated with a cocktail containing phorbol 12-myristate 13-acetate (PMA), ionomycin, Brefeldin A, *etc*. (eBioscience, 004975) for 5 h in the cell culture incubator at 37 °C. Subsequently, Fc block solution was added to block non-specific Fc-mediated interactions. Cells were stained for surface markers with monoclonal antibodies against CD4 (11-0041-82), CD25 (12-0251-81), and then fixed and permeabilized with Fixation/Permeabilization solution (eBioscience, 005523). Finally, intracellular/intranuclear cytokines were stained with monoclonal antibodies against IL-10 (12-7101-82), IL-17A (17-4321-81) and Foxp3 (17-5773-80), followed by flow cytometry analysis (BD FACSVerse^TM^, USA). All the antibodies were purchased from eBioscience.

### Enzyme-linked immunosorbent assay (ELISA)

The concentrations of TNF-α, IL-6, IL-1β, PGE2, MPO, IL-10, and IL-17A were determined for cell culture supernatant, serum and homogenized colon tissue using ELISA kits according to the manufacturer's instructions (Nanjing Jiancheng Bio, Nanjing, CN). In brief, RAW264.7 cells were pretreated with various concentrations of mEVs for 8 h followed by LPS stimulation for 20 h, then the supernatant was collected for ELISA analysis. The absorbance of each sample was detected at 450 nm by Multiskan FC.

### Quantitative Real-time PCR

Total RNA was extracted by Trizol Reagent (TIANGEN Biotech). 1 μg of total RNA was converted into cDNA with High-Capacity cDNA Reverse Transcription Kit (Applied Biosystems). Gene expressions of TNF-α, IL-6, IL-1β, IL-2, IL-10, IL-22, TLR-4, MyD88, iNOS, NLRP3 and GAPDH were quantitated with SYBR Green Master Mix (Applied Biosystems) using a Step One Plus TM Real-Time PCR Instrument (Applied Biosystems). GAPDH was used as a housing gene. Primers were designed and included in Supplementary Table S4.

### Western blot analysis

The cell nuclear and cytoplasmic proteins were extracted by nuclear and cytoplasmic protein extraction kit (Beyotime Biotechnology, CN). For tissue immunoblotting, total protein was extracted from colonic tissues using RIPA lysis buffer containing protease and phosphatase inhibitor (Beyotime Biotechnology, CN). The mEV proteins were extracted using the RIPA buffer (Beyotime Biotechnology, CN). The protein concentration of mEVs was determined by BCA assay kit. An equal amount of protein was subjected to SDS-PAGE and electro-transferred to a PVDF membrane. The membrane was immunodetected with specific antibodies overnight at 4 °C. Proteins were quantified by Image J software (NIH, Bethesda, MD). Antibodies against TSG101 (ab225877, Polyclonal), Alix (ab186429, Monoclonal), Calnexin (ab227310, Polyclonal), TLR-4 (ab13556, Polyclonal), MyD88 (ab219413, Monoclonal), IκBα (ab76429, Monoclonal), p-IκBα (ab133462, Monoclonal), COX-2 (179800, Monoclonal), iNOS (178945, Monoclonal), p65 (ab16502, Polyclonal), p-p65 (ab76302, Monoclonal), NLRP3 (ab210491, Monoclonal), Pro-caspase-1 (ab179515, Monoclonal), ASC (ab180799, Polyclonal) and HDAC1 (ab109411, Monoclonal) were purchased from Abcam (Cambridge, UK). β-actin (4967S, Monoclonal) was purchased from Cell Signaling Technology (Danvers, MA). CD9 (GTX76185, Monoclonal) and CD81 (GTX101766, Polyclonal) were purchased from GeneTex (South California, USA).

### Histology and immunohistochemistry

The colon segment was fixed using 4% paraformaldehyde and embedded in paraffin. The tissue was cut into 5 μm thick slices, and subjected to H&E and picrosirius red staining. For immunofluorescence analysis, antigens were retrieved by boiling tissue section in sodium citrate buffer, and endogenous peroxidase activity was blocked by incubation in 3% hydrogen peroxide solution. The tissue sections were incubated with 3% BSA for 30 min at room temperature to block non-specific binding, followed by straining with the primary antibodies overnight at 4 °C. After the secondary antibody incubation, the sections were stained with DAB and hematoxylin, and visualized under a light microscope (Olympus).

### Microbiota 16S rRNA gene sequencing

The genomic DNA of feces was extracted using the DNeasy PowerSoil Kit (QIAGEN, Inc., Netherlands). The bacterial 16S rRNA genes V4-V5 region was amplified using the forward primer 515F (5'-GTGCCAGCMGCCGCGGTAA-3') and the reverse primer 907R (5'-CCGTCAATTCMTTTRAGTTT-3'). Sample-specific 7-bp barcodes were incorporated into the primers for multiplex sequencing. PCR amplicons were purified, quantified and pooled in equal amounts. The paired-end 2×300 bp sequencing was performed using the Illumina MiSeq platform with MiSeq Reagent Kit v3 at Shanghai Personal Biotechnology Co., Ltd (Shanghai, China). The high-quality sequences were clustered into operational taxonomic units (OTUs) at 97% sequence identity by UCLUST 22. Sequence data analyses were mainly performed using QIIME and R packages (v3.2.0). The taxonomy compositions and abundances were visualized using MEGAN and GraPhlAn. LEfSe (Linear discriminant analysis effect size) was performed to detect differentially abundant taxa across groups using the default parameters.

### Statistical analysis

Data were presented as mean ± SD. One-way analysis of variance (ANOVA) was performed using SPSS 22 (SPSS Inc., Chicago, IL). *P* values < 0.05 were considered statistically significant (*p < 0.05, ^#^*p* < 0.01,^ ‡^*p* < 0.001). All other statistical tests were performed using the GraphPad Prime 8 (GraphPad Software, San Diego, CA, USA).

## Results

### Proteomic and miRNA functional analysis of mEVs

**Figure 1:**
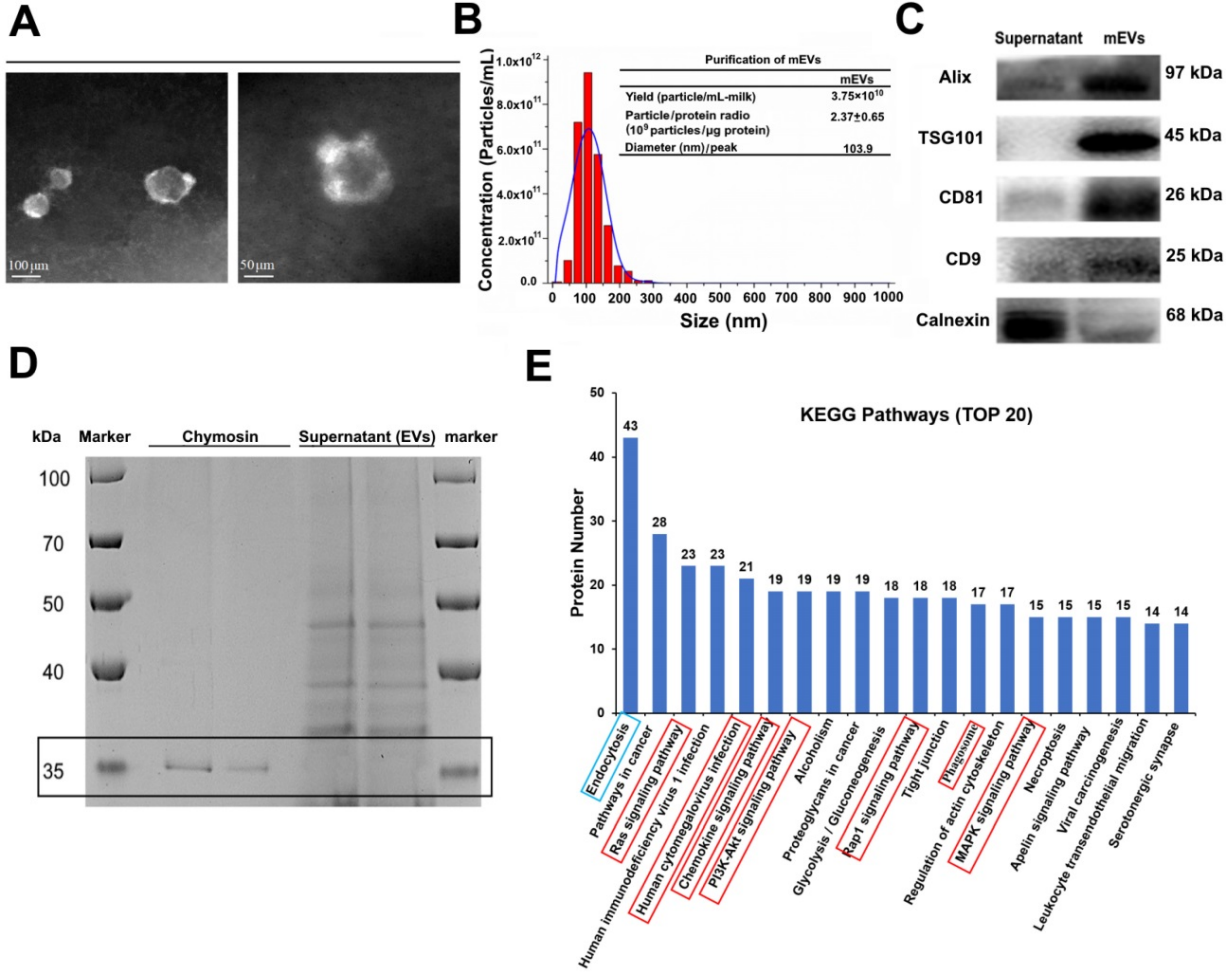
** Isolation and characterization of milk-derived extracellular vesicles (mEVs).** (A) Transmission electron microscopy images of mEVs. Scale bars represent 100 nm (left) and 50 nm (right). (B) Size distribution and purify analysis of mEVs by NTA. (C) mEV markers and calnexin analyzed with western blotting. (D) SDS-PAGE analysis showing removal of chymosin from the supernatant (containing mEVs) obtained after centrifugation at 16,500 g for 30 min. Molecule weight of chymosin is 30-45 kDa. (E) KEGG pathway analysis of mEV proteins (Top 20).

Bovine milk is an extremely complex matrix. It is difficult to isolate and purify mEVs using traditional ultracentrifugation. We obtained mEVs *via* an effective approach (chymosin treatment combined with ultracentrifugation and ultrafiltration) reported in our previous study 19. As shown in Figure 1, isolated mEVs were typically spherical in shape (Figure 1A), and their size ranged from 30 nm to 200 nm (Figure 1B). In addition, mEVs contained abundant EV-related proteins, including tetraspanins (CD9, CD81), ESCRT-I/II/III (TSG101), heat shock proteins (HSP70, HSP90), MHC class I, Alix, and Rab proteins, but only a marginal amount of the endoplasmic reticulum chaperone protein calnexin (Figure 1C, Table S1). Furthermore, chymosin (MW: 30-45 kDa) was removed *via* centrifugation at 16,500 g for 30 min (Figure 1D, black box). More importantly, we found that chymosin did not affect the integrity of mEV membrane proteins during the purification process 19.

To explore the function of mEVs, Gene Ontology (GO) annotations and KEGG pathway analysis were used to mine data of mEVs proteome. GO annotations showed that most proteins were involved in biological processes and cellular components (Figure S1). Furthermore, 507, 416 and 281 proteins were predicted to be involved in the immune-related response/process, cellular process and catalytic activity, respectively (Supplementary file 3). The top 20 KEGG pathways were presented in Figure 1E, and eight pathways were involved in inflammatory signaling (red boxes), including human cytomegalovirus infection, PI3K/Akt signaling pathway, chemokine signaling pathway, MAPK signaling pathway, and so on. Notably, 223 of 679 proteins were involved in the inflammatory signaling pathways, including NOD-like receptor signaling pathway, Toll-like receptor signaling pathway and NF-κB signaling pathway (Table S2).

A total of 678 miRNAs were identified in mEVs. We found that 36 of the top 100 miRNAs targeted inflammatory pathways, including 10 miRNAs implicated in IBD (Table S3, Supplementary file 2). The top 5 miRNAs implicated in IBD included miR-148a (1^st^ in all microRNA), miR-27b (15^th^), miR-152 (48^th^), miR-10174-3p (49^th^) and miR-182 (62^th^), which referred to the NOD, TLR4 and T cell receptor signaling pathways (Figure S2). In fact, those miRNAs have been reported to play crucial roles in immune inflammatory diseases 23, 24.

### The immunomodulatory effects of mEVs *in vitro*

To evaluate the cellular uptake of mEVs *in vitro*, PKH26-labeled mEVs or free dye PKH26 were added and incubated with RAW264.7 cells. Compared with free dye PKH26, PKH26-labeled mEVs were internalized by RAW264.7 cells. As shown in Figure S3A, mEVs were mainly located in the cytoplasm. Next, the dose-dependence and time-dependence of mEV uptakes were evaluated. We observed that the uptake of mEVs increased with increasing concentration of mEVs and reached the plateau at 200 μg/mL (Figure S3B and S3D left). Similarly, a time-dependent increase in mEVs uptake was observed within 8 h, and the uptake reached a plateau at 16 h (Figure S3C and S3D right). These results demonstrated that mEVs could be internalized by RAW264.7 cells in a dose- and time-dependent manner.

Based on the *in vitro* uptake data, the appropriate concentrations (30, 120, and 480 μg/mL) of mEVs and incubation time (8 h) were selected to evaluate the immunomodulatory effect of mEVs on RAW264.7 cells. As shown in Figure S4B, mEVs did not affect the cell viability at 480 μg/mL. The cellular inflammatory model was established by 100 ng/mL LPS, which significantly increased the production of nitric oxide (NO) and prostaglandin E2 (PEG2) in cells and changed cellular morphology, such as the spherical M0 macrophages were flattened into pancake-like M1 macrophages. Interestingly, mEVs inhibited the release of NO and PEG2, and effectively suppressed the polarization transition of macrophages (Figure S4C-D and Figure S5). Furthermore, mEVs attenuated the production of various cytokines at both protein and mRNA levels (Figure S4E-J).

**Figure 2:**
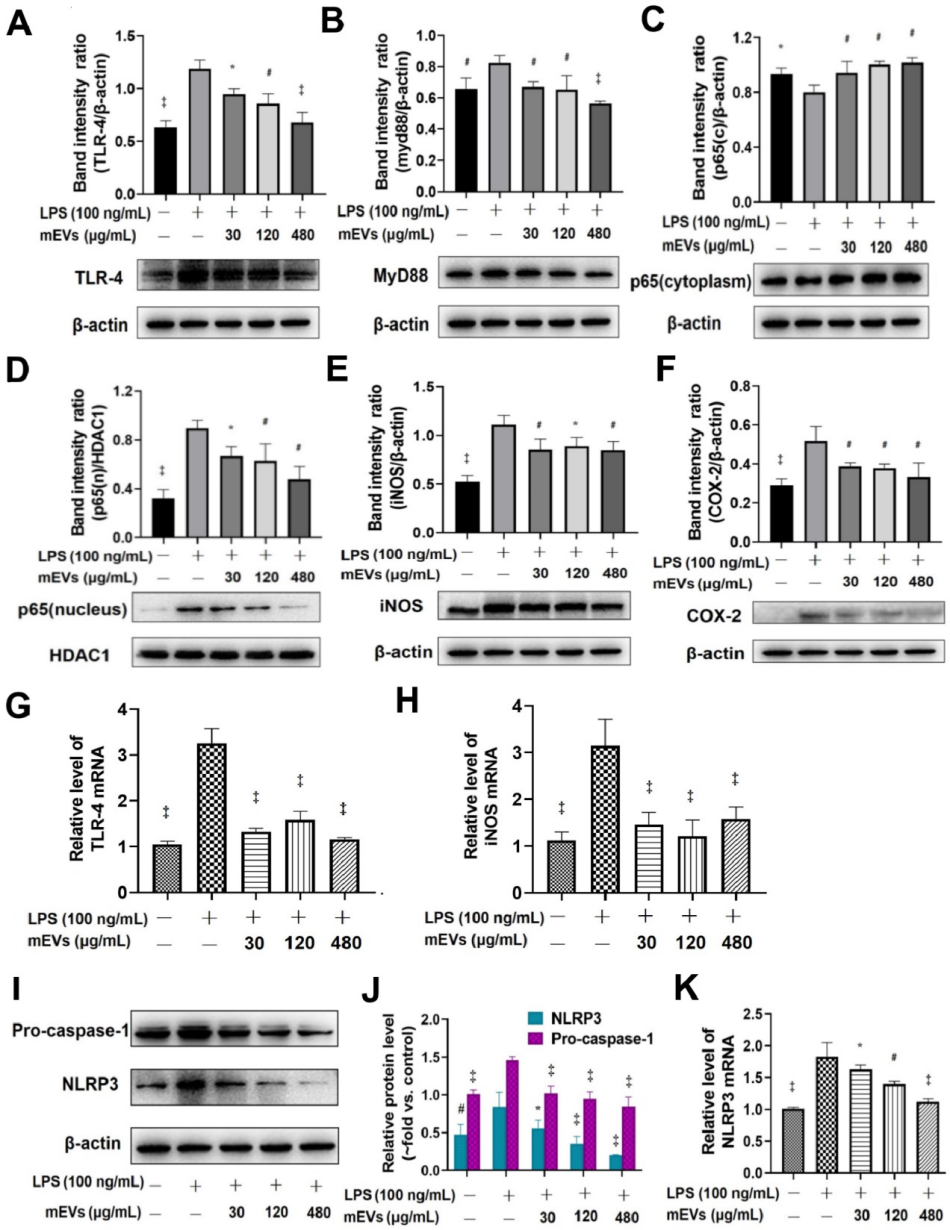
**mEVs inhibit NF-κB signaling and NLRP3 inflammasome activation *in vitro*.** (A-F) Effects of mEVs on TLR4-NF-κB signaling pathway in RAW264.7 cells. (G-H) mRNA expression levels of TLR4 and iNOS in RAW264.7 cells. (I-K) Analysis of NLRP3 inflammasome activation in mEVs-treated RAW264.7 cells. Proteins levels were quantified by normalization to β-actin or HDAC1. Data were presented as mean ± SD of three independent experiments. ^*^p < 0.05, ^#^*p* < 0.01 and ^‡^*p* < 0.001 compared LPS model groups.

To further explore the immunomodulatory mechanism of mEVs, two classical inflammatory signaling pathways, TLR4-NF-κB and NLRP3, were investigated based on the bioinformatics of mEV proteome and miRNAs (Figure 2). Compared with LPS group, mEVs downregulated the protein levels of TLR4 and Myd88 in a dose-dependent manner (Figure 2A-B). The expression of p65 protein was markedly increased in the nucleus and decreased in the cytoplasm after LPS stimulation, while mEVs reversed the cellular distribution of p65 in the nucleus and the cytoplasm (Figure 2C-D). These results indicate that mEVs could inhibit the translocation of p65 into the nucleus and thereby suppress the activation of NF-κB signaling pathway. Moreover, the expression of NF-κB downstream protein inducible NO synthase (iNOS) and cyclooxygenase-2 (COX2) was inhibited by mEVs in LPS-stimulated RAW264.7 cells (Figure 2E-F). This at least partially explains why mEVs could effectively suppress the release of NO and PEG2 (Figure S4). In addition, the expression of pro-caspase-1 and NLRP3 in the NLRP3 signaling pathway, which was up-regulated in LPS-induced cells, was also reversed by mEVs in a dose-dependent manner (Figure 2I-K).

### Biodistribution of mEVs in DSS-induced ulcerative colitis

Before testing the bioactivities of mEVs *in vivo*, we compared the biodistribution of mEVs following oral or intravenous administration. To do so, mEVs were labeled with the near-infrared fluorescent dye DiR 25. DiR-labeled mEVs were administered to C57BL/6 mice by oral gavage, and mouse organs (small intestines, colon, liver, spleen, and kidneys) were collected and imaged at 0, 1, 6 and 12 h post administration. As shown in Figure S6A, mEVs reached the small intestines at 1 h and the colon at 6 h. After 12 h, mEVs were mainly located in the colon. There was no apparent accumulation of mEVs in other organs. To rule out the possible accumulation of free dye *per se*, free DiR was administrated to mice as a negative control. As shown in Figure S6B and S6C, compared with DiR-mEVs, free dye DiR had reached the colon at 1 h and disappeared in all organs from 6 h onward, suggesting that the free dye was discharged from the body through feces. These findings demonstrate that the free dye does not affect the distribution of mEVs, and that mEVs *via* oral administration can reach the colon and stay for a long time in the gut. In contrast, mEVs administered *via* tail vein injection accumulated predominantly in the liver and spleen (Figure S7). These findings suggest that the biological effects of mEVs could differ depending on the delivery routes.

### mEVs restore gut immunity in DSS-induced ulcerative colitis

**Figure 3:**
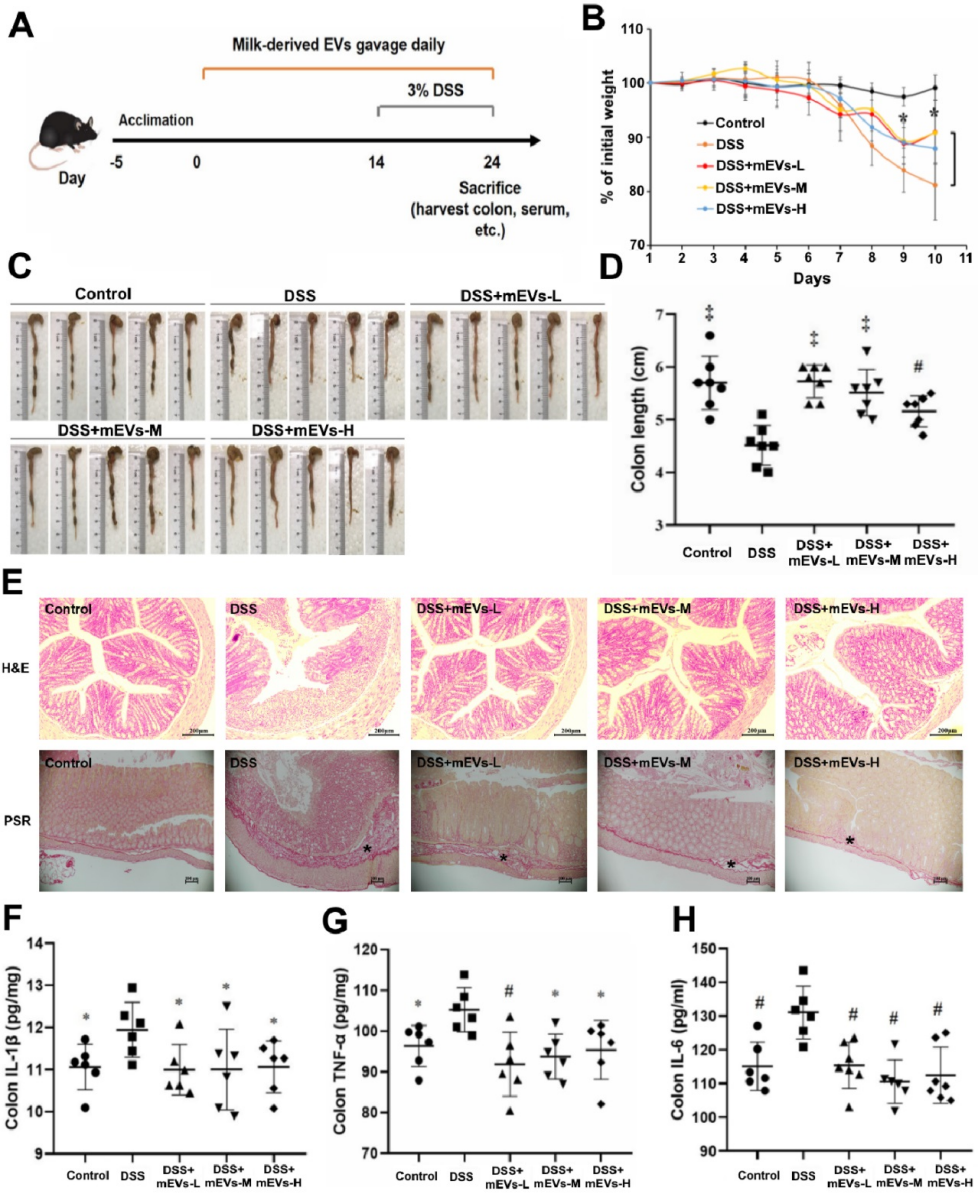
**mEVs alleviate DSS-induced ulcerative colitis (UC).** (A) A schematic diagram illustrating administration schedule of mEVs in a DSS-induced mouse model of UC. (B) Change of body weight among different treatment groups. ^*^p < 0.05 *vs.* the DSS group. (C, D) Colon length comparison. (E) Representative colon sections stained with hematoxylin and eosin (H&E, Scale bars represent 200 μm) and picrosirius red (PSR, Scale bars represent 100 μm). Asterisks indicate fibrosis within the intestinal submucosal layer. (F-H) Protein levels of colonic IL-1β, IL-6, and TNF-α determined by ELISA. Data were presented as mean ± SD (n = 7). ^*^p < 0.05, ^#^*p* < 0.01 and ^‡^*p* < 0.001 *vs.* DSS group (UC).

The immunomodulatory effects of mEVs *in vivo* were tested in a mouse colitis model induced by DSS. As expected, gradual weight loss occurred in DSS-treated mice. In contrast, mEVs treatment markedly prevented body weight loss (Figure 3B) and shortening of colon length (Figure 3C-D) in DSS-induced UC mice. Furthermore, mEVs, as demonstrated by H&E and picrosirius red staining, attenuated intestinal epithelium disruption, infiltration of inflammatory cells and generation of fibrotic tissues in UC (Figure 3E). Cytokine disorder is one of the key features of colitis and the imbalance between proinflammatory and anti-inflammatory cytokines that occurs in IBD impedes the resolution of inflammation 26. A significant increase in various cytokines was observed in the serum and colonic tissue of DSS-treated mice. In contrast, mEVs treatment inhibited DSS-induced upregulation of IL-1β, TNF-α, IL-6, IL-2, and IL-22 (Figure 3F-H and Figure S8). In addition, the activity of myeloperoxidase (MPO), a pivotal marker of inflammation and oxidative stress in the colon 27, was markedly increased in DSS-treated mice (Figure S8D). However, treatment with mEVs suppressed the elevation of MPO activity in DSS-treated mice. These results indicate that mEVs could prevent mouse colitis *via* inhibition of the pro-inflammatory cytokine production.

**Figure 4:**
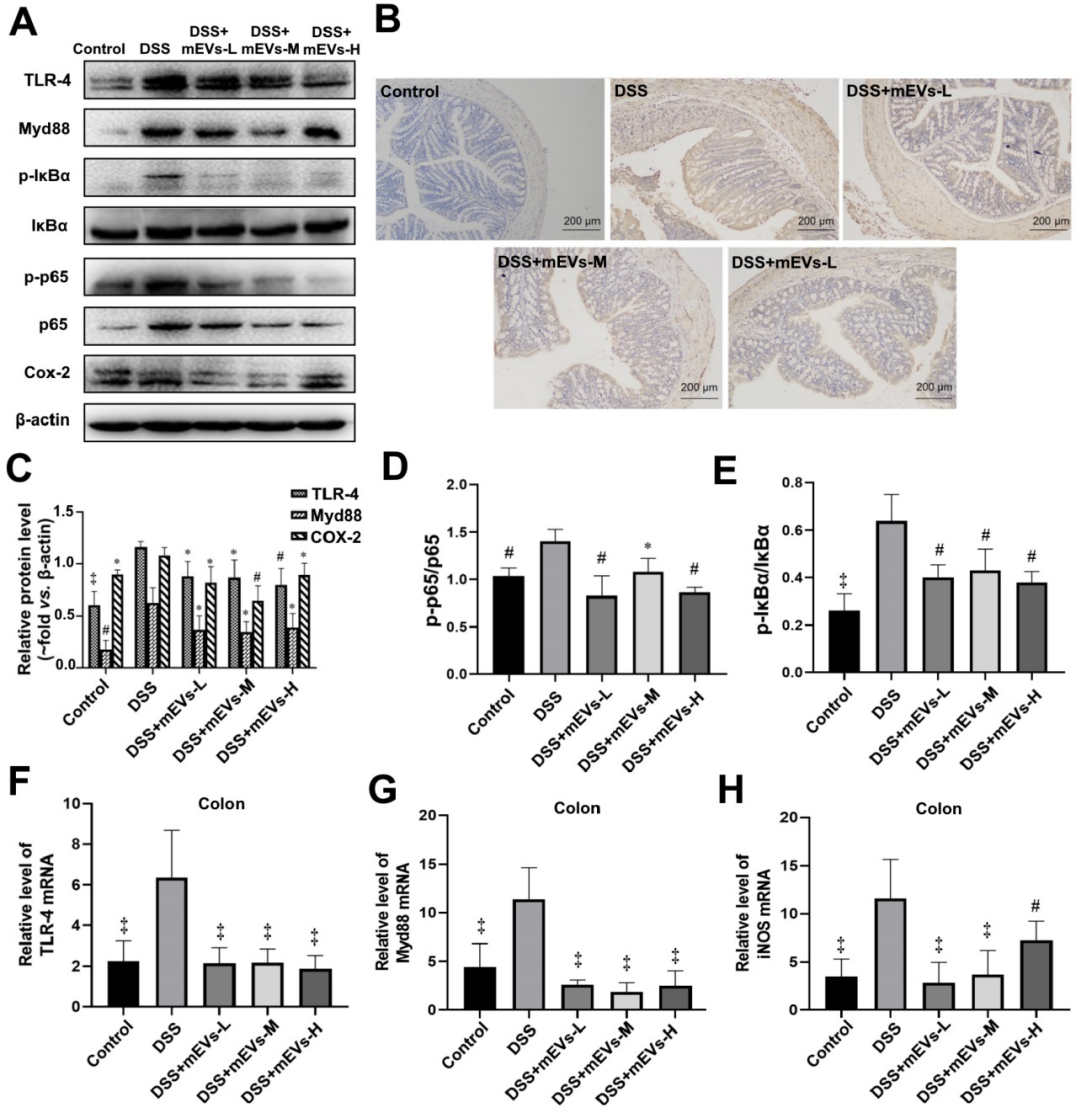
** mEVs inhibit TLR4-NF-κB signaling pathway *in vivo*.** (A) Representative Western blotting of TLR4, Myd88, IκBα, p65, cox2, phosphorylated IκBα (p-IκBα), p-p65, and β-actin in the colon. (B) Expression of NF-κB p65 in colon tissue analyzed by immunohistochemistry. (C-E) Quantification of the protein expression levels of TLR4, Myd88, Cox2, p-IκBα and p-p65, normalized to β-actin. (F-H) Gene expression levels of TLR4, Myd88 and iNOS in colon tissue. GAPDH was used as a housing gene for normalization of mRNA levels. Data were presented as mean ± SD (n = 7 per group). ^*^p < 0.05, ^#^*p* < 0.01 and ^‡^*p* < 0.001 *vs.* DSS group (UC).

**Figure 5:**
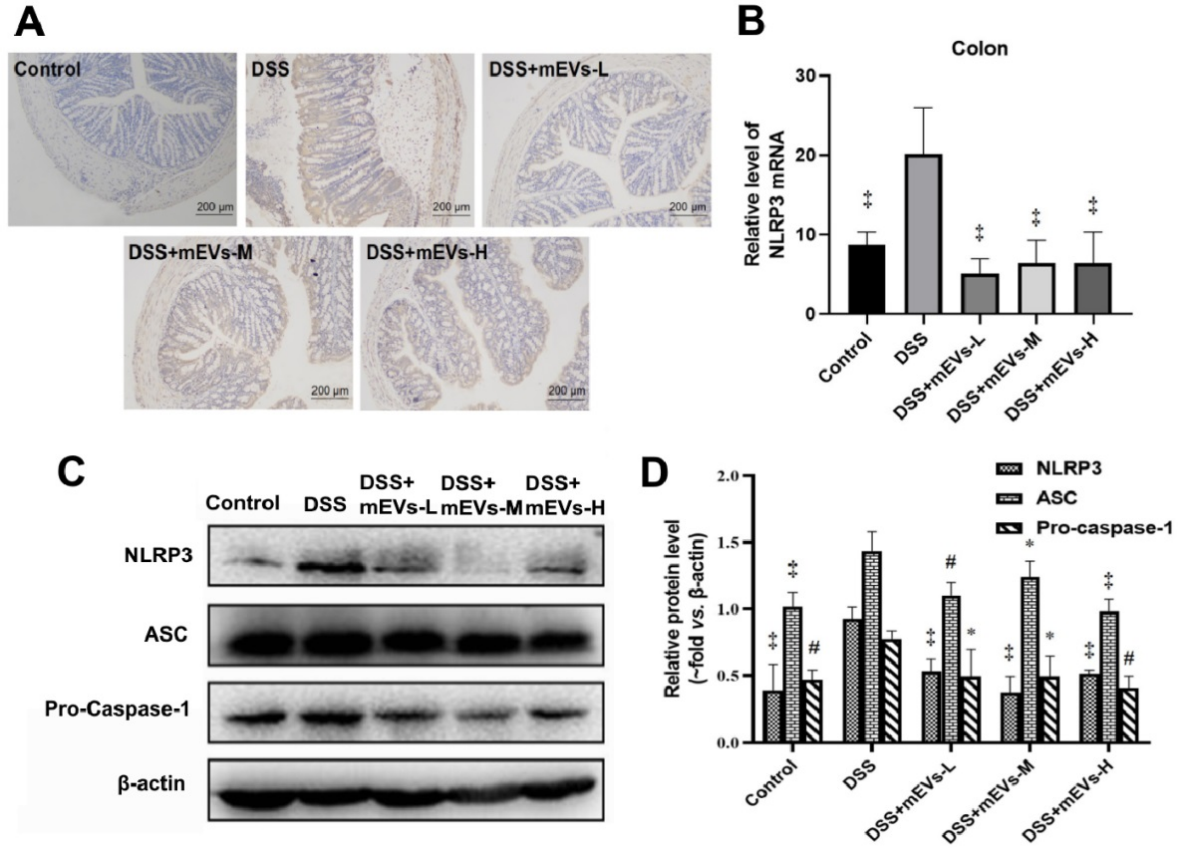
** mEVs inhibit NLRP3 inflammasome activation *in vivo*.** (A) Representative colon sections for NLRP3 by immunohistochemistry. (B) mRNA expression levels of colonic NLPR3 determined by qPCR. (C) Proteins levels of NLRP3 inflammasome components in colon tissues analyzed by Western blotting. (D) Quantification of NLRP3 inflammasome components per Western blotting analysis in panel C. Protein levels were normalized to β-actin. Data were presented as mean ± SD (n = 7 per group). ^*^p < 0.05, ^#^*p* < 0.01 and ^‡^*p* < 0.001 *vs.* DSS group.

To determine the immune regulatory mechanism of mEVs in DSS-induced colitis model, we examined the expression of several crucial regulators in the TLR4-NF-κB and NLRP3 signaling pathways by western blotting, immunohistochemistry and RT-PCR analysis. As shown in Figure 4A-E, expression levels of TLR4, Myd88, COX2, phosphorylated IκBα (nuclear factor of kappa light polypeptide gene enhancer in B-cells inhibitor, alpha) and p65 proteins were markedly increased in DSS-treated mice, however, the changes of those TLR4-NF-κB signaling pathway components were effectively inhibited upon treatment with mEVs. In consistence, the up-regulation of TLR4, Myd88 and iNOS in DSS-treated mice was inhibited at mRNA level by mEV treatment (Figure 4F-H). Similar to the TLR4-NF-κB signaling pathway, the increase in the expression of the NLRP3 signaling pathway key components, including NLRP3, apoptosis-associated speck-like protein (ASC), and pro-caspase-1, in DSS-treated mice, was significantly attenuated upon treatment with mEVs (Figure 5). These findings suggest that mEVs could suppress TLR4-NF-κB and NLRP3 signaling pathway and thus prevent mouse colitis.

**Figure 6:**
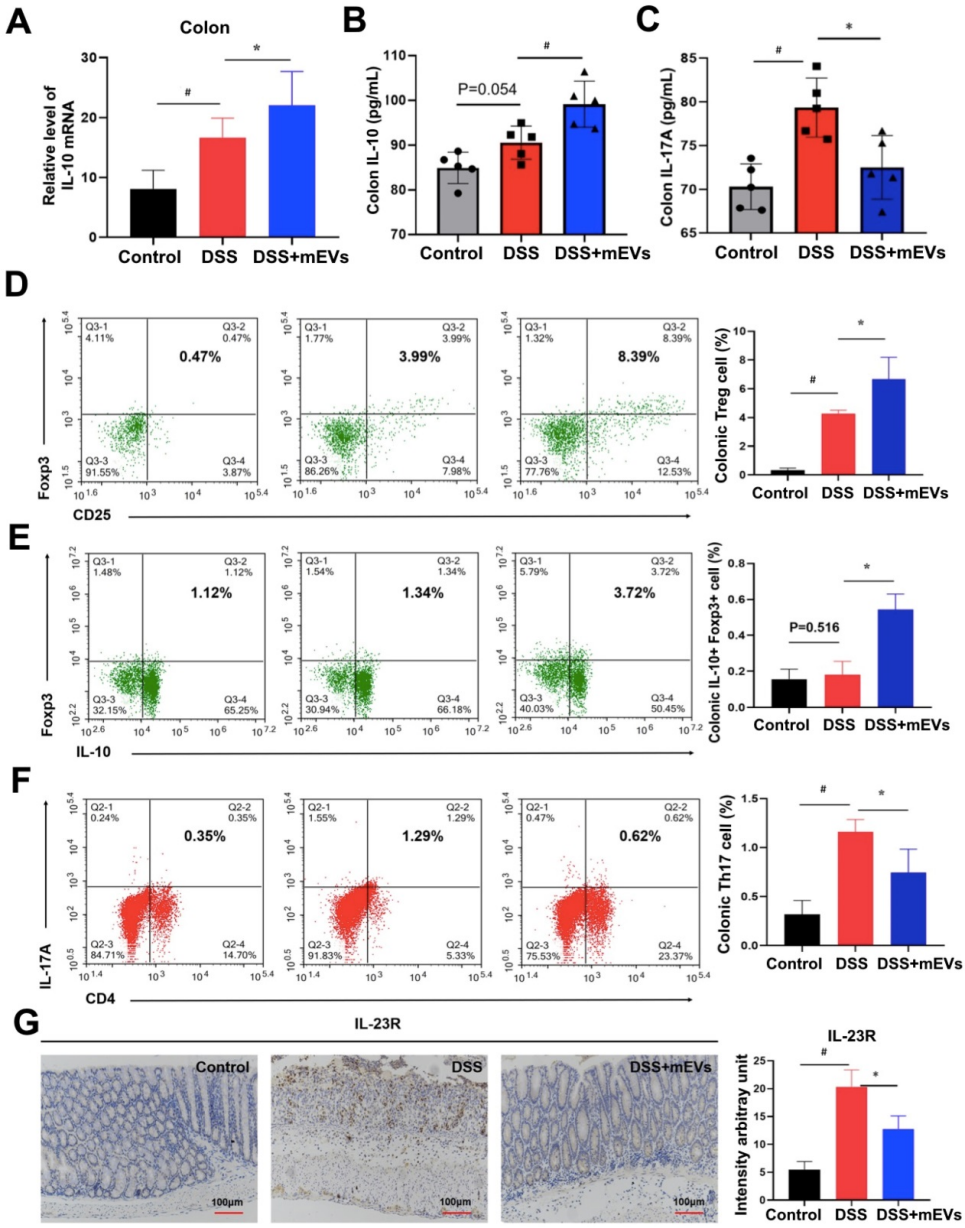
** mEVs restore DSS-disturbed balance between Treg and Th17 cells in the colonic lamina propria (LP).** (A) mRNA expression of IL-10 in colon tissue (normalized to GAPDH). (B, C) Protein levels of cytokines IL-10 and IL-17A in colon tissue measured by ELISA. (D-F) Flow cytometry analysis of CD4^+^CD25^+^FoxP3^+^ Treg cells (D), IL-10^+^FoxP3^+^ cells (E), and Th17 cells (F) in the colonic LP. (G) Expression of IL-23R protein in the colon. Data were presented as means ± SD. N = 4 to 5 mice per group. ^*^ *p* < 0.05, ^#^ *p* < 0.01.

Given that IL-10 is a major product of Treg cells and plays a crucial role in Treg cell-mediated colitis alleviation 28, and that the pathogenic roles of IL-17A, IL-22, and IL-2 produced by Th17 cells have been well documented in IBD 29, we examined the alteration in cytokine production and Treg/Th17 balance in the intestinal mucosa. Indeed, expression of a broad range of cytokines was altered in DSS-treated mice, which was largely restored by mEV treatment (Figure 6A-C, Figure S8). Interestingly, administration of mEVs markedly increased the expression of IL-10 while deceased that of IL-17A in DSS-treated mice. Next to this, we analyzed the Treg/Th17 balance in intestinal mucosa by flow cytometry. As shown in Figure 6D, mEVs increased the number of Treg cells in the colonic lamina propria (LP) of the DSS-induced colitis model (*P* < 0.05). In particular, the number of IL-10^+^Foxp3^+^ Treg cells also increased in DSS+mEVs-L group compared to DSS group (Figure 6E). Furthermore, expression of Th17A in CD4^+^ T cells compartment in DSS+mEVs-L group was significantly lower than that in DSS group (Figure 6F). Of note, expression of IL-23R, which is closely associated with IL-17A production in inflammation 30, was also inhibited by mEVs in DSS-treated mice (Figure 6G). Taken together, mEVs restored intestinal homeostasis by re-balancing the Treg/Th17 cell populations and production of proinflammatory and anti-inflammatory cytokines in a mouse model of UC.

### mEVs rectify gut microbiota imbalance in ulcerative colitis

**Figure 7:**
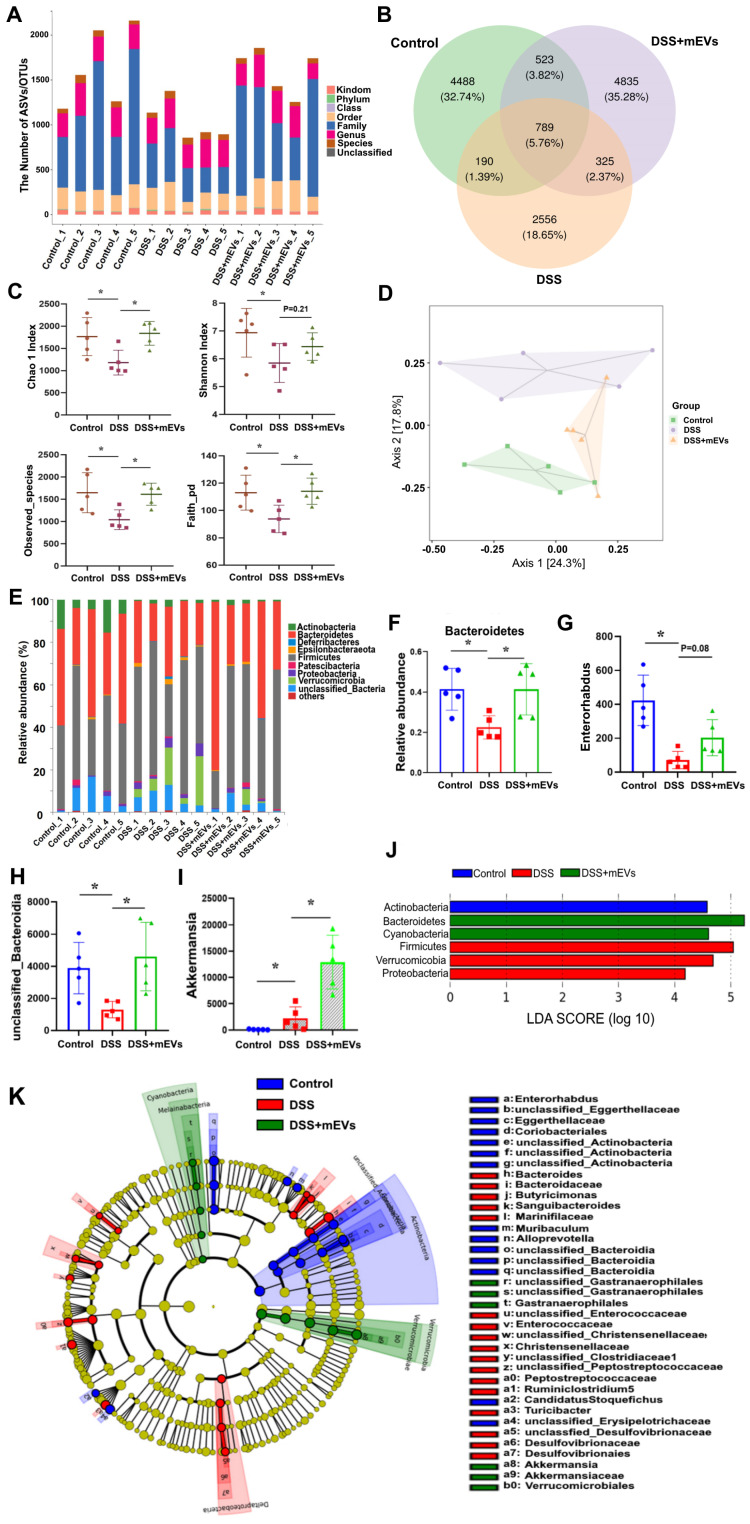
** mEVs reshape the gut microbiota composition in DSS-induced UC mice.** Mouse feces were collected two days prior to euthanasia. Mice were treated according to the schedule illustrated in Figure 3A. Bacterial DNA from feces was analyzed using 16S rRNA gene sequencing (n = 5). (A) Results of the taxonomic annotation. (B) Venn diagram of ASV/OTU in the feces. The numbers of ASVs/OTUs were evaluated by 16S rRNA gene V4-V5 pyrosequencing reads. ASVs, Amplicon Sequence Variants; OTUs, Operational Taxonomic Units. (C) Alpha diversity indexes calculated with QIIME2 according to ASV/OTU numbers of each group. ^*^p < 0.05. (D) β-diversity evaluated using the weighted UniFrac-based PCoA. (E) Bar graphs showing the relative abundance of different bacteria at the phylum level. (F) Relative abundances of* Bacteroidetes* at the phylum level. ^*^p < 0.05. (G-I) Changes of the OUTs of *Enterorhabdus*, *unclassified_Bacteroidia,* and *Akkermansia* at the genus level. ^*^p < 0.05. (J) Linear discriminant analysis (LDA) effect size (LEfSe) method was used to investigate bacterial community at the phylum level. LDA score higher than 3 indicates a higher relative abundance in the corresponding group than that in other groups. (K) Cladogram based on LEfSe analysis showing community composition of the gut microbiota in mice.

Imbalance of gut microflora is considered a key factor in DSS-induced colitis 31, 32. Having established the immunomodulatory effect of mEVs, we asked if mEVs could influence gut microbiota in DSS-induced colitis. To address this query, we analyzed the feces from three groups of mice (Control, DSS, and DSS+mEVs-L group) by 16S rRNA sequencing. We found that the gut microbial diversity decreased in DSS-induced colitis, which was moderately restored upon mEVs treatment (Figure 7). Specifically, a lower number of OTU was observed in DSS-treated mice compared with that in control or mEVs-treated mice (Figure 7A-B).

The α-diversity indices evaluating gut microbial community richness and community diversity, including Chao 1 index, Shannon index, Observed_species, and Faith_pd were all significantly decreased in DSS-treated mice, whereas treatment with mEVs restored the α-diversity of gut microbiota effectively (Figure 7C). The PCoA analysis showed that mEVs could shape the gut microbiota in DSS-treated mice and restore them to a normal microbial community (Figure 7D).

**Figure 8:**
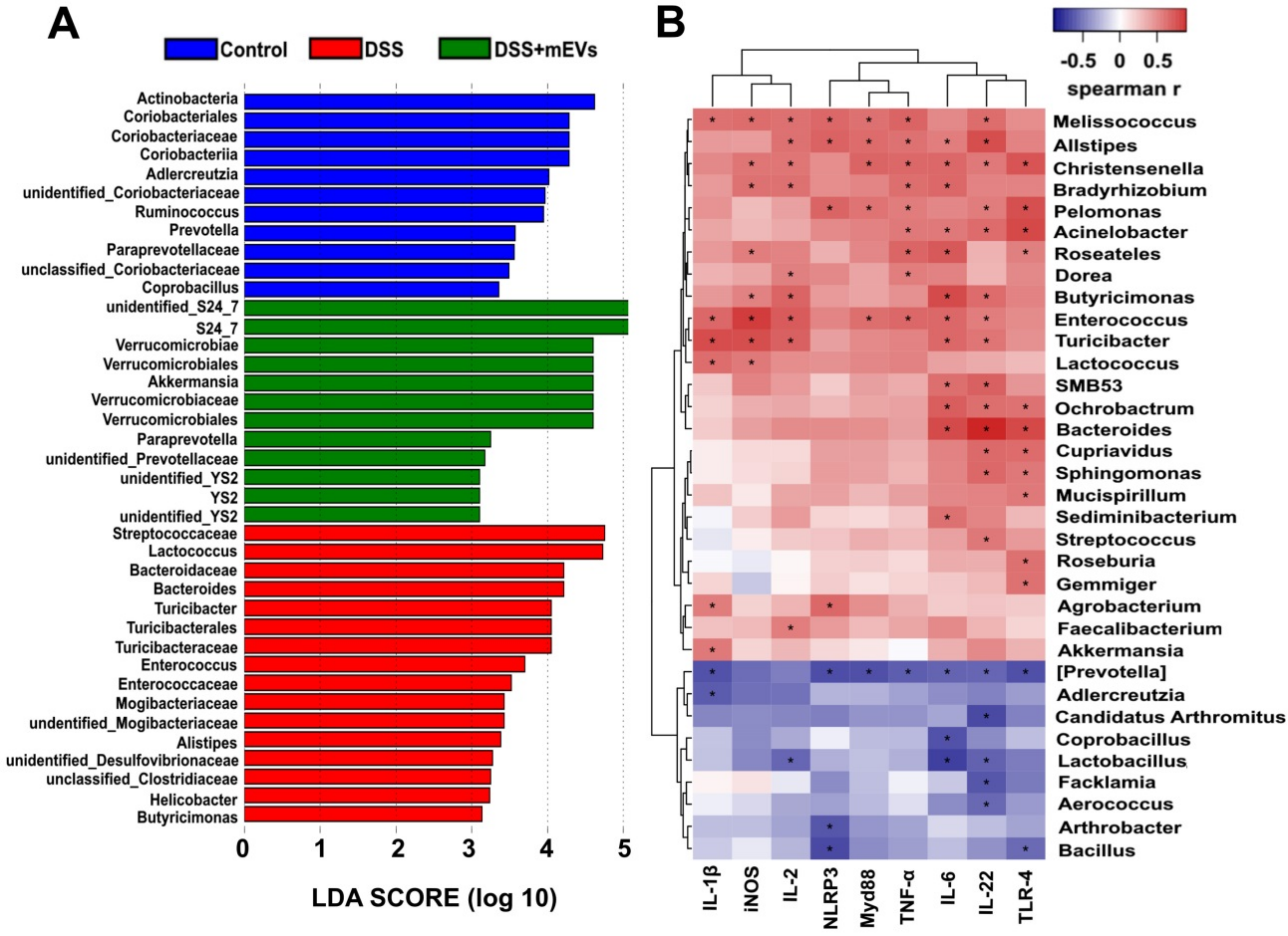
** Correlation analysis between the gut microbiota and intestinal immune inflammatory factors.** (A) Differentially enriched gut microbiota in each group of mice at the genus level by linear discriminant analysis (LDA). LDA score higher than 3 indicates a higher relative abundance in the corresponding group than that in other groups. (B) Correlation matrix showing the strength of correlation between the gut microbiota (at the genus level) and the concentrations of immune inflammatory factors in the colon. Values in cells are Spearman correlation coefficient. Statistical significance was determined for all pairwise comparisons using Spearman's method. *p* < 0.05. Spearman r values range from -0.5 (blue) to 0.5 (red).

The relative abundance of bacteria, similar to the findings by Sartor *et al.* 33, was severely disturbed in DSS-treated mice, with the relative abundance of phyla *Bacteroidetes* being significantly decreased while that of the phyla *Proteobacteria* and *Verrucomicrobia* being markedly increased (Figure 7E-F, 7J). Strikingly, the relative abundance of bacteria was recovered nearly to the level in the control mice (Figure 7E-F). At the genus level, DSS-treated mice displayed a depletion of *Enterorhabdus* and *unclassified_Bacteroidia*, which was partially recovered by mEVs treatment (Figure 7G-H). Similar to a recent report 34, the taxonomic branches of *Enterococcaceae* and *Desulfovibrionales-unclassified Desulfovibrionaceae* were significantly increased in the DSS-induced mice. In contrast, these pathogenic bacteria remained unchanged in EVs-treated group (Figure 7K). Interestingly, a promising probiotic, *Akkermansia* was significantly increased in EVs-treated mice (Figure 7I and 7K, Figure 8A). These results indicate that mEVs could shape the gut microbiota in DSS-induced colitis.

### mEVs may restore gut immunity by reshaping gut microbiota in ulcerative colitis

To explore how mEVs modulate intestinal immune and gut microbiota in colitis, we conducted a correlation analysis between gut microbiota and immune-inflammatory factors by Spearman's rank correlation method. We found that the increase in harmful bacteria, such as *Enterococcus*, *Turicibacter*, *Helicobacter*, *Desulfovibrionaceae*, *unclassified Desulfovibrionaceae*, *Mogibacteriaceae*, was positively correlated with expression of proinflammatory cytokines and key genes of the immune-inflammatory pathways in DSS-induced colitis (Figure 8 and Figure S9). In contrast, the decrease in beneficial bacteria, such as *Akkermansia*, *S24_7*, *Paraprevotella* and *Verrucomicrobiaceae*, was negatively correlated with the expression of proinflammatory cytokines and key genes of the immunomodulatory pathways (Figure 8 and Figure S9). These findings suggest that mEVs could regulate intestinal immune homeostasis* via* gut microbiota, and consequently prevent mouse colitis.

## Discussion

EVs are present in milk (mEVs) and play a critical role in the development of immune system 35. In this study, we comprehensively investigated the therapeutic effects of mEVs on ulcerative colitis and potential mechanisms therein. We demonstrated that mEVs contain abundant proteins and microRNAs that are involved in immune regulatory pathways. Accordingly, mEVs inhibited inflammatory responses mediated by TLR4-NF-κB signaling pathway and NLRP3 signaling pathway, both *in vitro* and in a mouse model of UC. Oral administration of mEVs alleviated mouse UC by restoring gut cytokine homeostasis, immune cell balance between IL10^+^Foxp3^+^ Treg cells and Th17 cells, and gut microbiota.

Breast milk contains various immune modulatory components, including immune-competent cells, lipids, proteins (including antibodies and peptides), and miRNAs, which provide immunity to the infant for infection prevention and immune system development 36, 37. Interestingly, recent studies also demonstrated the presence of immune-modulatory EVs in breast milk of various animal species, including rodents, pigs, pandas, bovines, and humans 38. For instance, human mEVs inhibit production of inflammatory cytokines (TNF-α, IL-2 and IFN-γ) in stimulated monocytes while increasing anti-inflammatory Foxp3^+^ Treg cells in peripheral blood *in vitro* 39. In addition, porcine mEVs can protect intestinal epithelial cells from apoptosis 10. In line with this, we now show that bovine mEVs enriched with immunomodulatory proteins and miRNAs inhibit cytokine production and macrophage polarization towards proinflammatory phenotype. These findings suggest that EVs derived from breast milk of various animal species and humans exert similar immunomodulatory effects although the relative activity of human mEVs and animal mEVs remains unclear. Given the easy access to bovine milk, despite the expression of proteins may differ in bovine milk from cow to cow and day to day, production of bovine mEVs can be scalable. Moreover, considering the high prevalence of bovine milk allergy (0.25-4.9% in general population but particularly higher in children) 40 and the importance of milk in human gut development, mEVs (which lack of allergens 19) or mEVs-based biologics may benefit patients allergic to bovine milk.

Despite the promising immunomodulatory function of mEVs, it remains challenging to identify the components that exert bioactivity of mEVs. With bioinformatic data analysis, we identified a large number of mEVs-associated proteins and miRNAs that were involved in immune signaling pathways, including NF-κB signaling pathway and NOD-like receptor signaling pathway. Intriguingly, 10 out of 678 miRNAs identified in mEVs were implicated in IBD. In particular, miR-148, the most abundant miRNA in mEVs, has been reported to inhibit NF-κB signaling pathway and suppress colitis and colitis-associated tumorigenesis 23. Although we were not able to demonstrate if the protective effects of mEVs in UC was solely due to miR-148, given the fact that miR-148 modulates antigen presentation of dendritic cells *via* TLR (including TLR4) signaling pathways 41 and mEVs inhibited TLR4-NF-κB signaling pathways both *in vitro* and *in vivo* in our study, it is reasonable to speculate that miR-148, at least partly, contributes to the immunomodulation function of mEVs. Among mEV proteins, in contrast to mEVs miRNAs, the proteins involved in endocytosis were the most abundant according to KEGG pathway analysis. This finding suggests that mEVs may be taken up by cells *via* endocytic pathways attributed to certain membrane proteins 42.

One important finding in this study is that mEVs *via* oral administration are able to reach the colon and ameliorate intestinal inflammation. It is known that TLR4, MyD88, and their downstream signaling molecules (IκBα and p65) play a critical role in the development of DSS-induced UC 43. So is NLRP3 inflammasome 44. In line with those pathological mechanisms of UC, in the current study, oral administration of mEVs could attenuate inflammatory response *via* inhibiting TLR4-NF-κB signaling pathway and NLRP3 inflammasome activation in the inflamed colon, and therefore restore cytokine homeostasis and protect mice from UC. These findings are supported by our bioinformatics data that abundant mEV cargoes could target these two inflammatory signaling pathways (Figure 1E and Table S2). To our knowledge, there was no prior report that mEVs regulate immune response *via* these two signaling pathways. In agreement with our findings, a very recent study reported that bovine milk P100K EVs (pellets obtained by 100,000 g ultracentrifugation for 1 h) alleviated colitis *via* restoring expression of A20 (or TNFAIP3, tumor necrosis factor alpha-induced protein 3) 45, an intracellular ubiquitin-editing protein that plays a key role in the negative feedback regulation of NF-κB signaling in response to multiple stimuli 46. Furthermore, blocking TLR4-NF-κB signaling pathway could regulate the differentiation and balance of the colonic Treg cell pool in colitis 6. Treg cells are suppressors of proinflammatory immune cells such as Th17 cells, and secrete anti-inflammatory cytokine IL-10 47. In this study, we noticed the imbalance between Treg (IL-10^+^Foxp3^+^) cells and IL-17A producing cells (Th17 cells) in UC, attributed to the increase in Th17 cells, as previously reported 48. Strikingly, oral administration of mEVs restored the Treg/Th17 cell balance in the intestinal mucosa. Accordingly, levels of IL-10 were increased while those of IL-17A, IL-22, and IL-23R secreted by Th17 cells were reduced in the colon. In consistence with a recent report 49, elevated levels of the general inflammation markers IL-1β, TNF-α and IL-6 in both serum and colon tissue of UC mice were effectively diminished by mEVs. At the cellular level, mEVs could suppress the production of proinflammatory cytokines and their downstream mediators including TNF-α, NO and PGE2 (Figure S4). Since the cytokines released by lamina propria immune cells are directly implicated in the pathogenesis of UC 26, the rectification of cytokine disorder may be the predominant mechanism of mEV treatment efficacy.

In addition, it has been reported that mEVs could protect necrotizing enterocolitis *via* modulating expression of Mucin 2 (MUC2) and abundance of MUC2^+^ goblet cells 50. Muc2 mucin is a major constituent of the mucosa layer on colonic epithelium, disruption of which will increase epithelial exposure to gut bacteria and cause severe colitis 51. In fact, our previous data showed that oral administration of mEVs could upregulate MUC2 expression and enhance intestinal immunity 19. Therefore, restoration of MUC2 expression may also partly contribute to the protective effects of mEVs in UC.

The gut microbiota is another crucial factor in the etiopathogenesis of UC 52. As previously reported 32, DSS-induced colitis caused an imbalance of gut microbiota, including an increase in pathogenic bacteria and a decrease in gut microbial diversity. Since we and others have demonstrated that mEVs could alter gut microbiome composition in the absence of gut inflammation 19, 53, we explored if mEVs could influence the gut microbiota in UC. Surprisingly, mEVs reshaped and restored the composition of DSS-disturbed gut microbiota in mice. In normal host, commensal bacteria could activate a continuous homeostatic response program through epithelial cells, macrophages, T lymphocytes and B cells 54. Microbiotas from human patients with IBD alter the balance of gut Th17 and RORγt^+^ regulatory T cells and exacerbate colitis in mice 55. The bacterial imbalance can cause exposure of host to microbial antigens, activate bacterial transmembrane pattern recognition receptors, and ultimately overwhelm immune tolerance. Those bacterial receptors mediate activation of central signaling cascades, including NF-κB, Akt and MAPK pathways 33, 54. Furthermore, the microbiota (non-pathogenic bacteria) or microbiota metabolites (such as short-chain fatty acids) can regulate NF-κB activation and dynamic balance of Treg/Th17 cells, and therefore prevent excessive inflammation 56. More importantly, there was a strong correlation between the gut microbiota and inflammatory cytokines or key genes of the immune inflammatory pathways (Figure 8 and Figure S9). These results suggest that mEVs may regulate intestinal immune homeostasis *via* the gut microbiota in both healthy and diseased mice, however, the exact mechanisms need further investigation.

Of particular interest, oral administration of mEVs increased the abundance of some beneficial gut microbes, such as *Akkermansia* (Figure 7I and Figure 8A). In fact, the relative abundance of *Akkermansia* in the gut microbiota has a clear negative correlation with intestinal inflammatory diseases 57, and treatment with *Akkermansia* was reported to ameliorate mucosal inflammation *via* microbe-host interactions 58. Whether *Akkermansia* can be used as probiotics for the treatment of UC needs more scientific evidence, and how mEVs increase the abundance of *Akkermansia* in the gut remains to be elucidated. Moreover, despite the fact that our mEV isolation method with chymosin increased the yield and purity of mEVs, our isolation process, like any other EV isolation methods, may enrich specific mEV subtypes while remove others. The biological significance of specific subtypes of mEVs in the gut needs further investigation.

To conclude, oral administration of mEVs attenuated intestinal inflammation *via* inhibiting TLR4-NF-κB and NLRP3 signaling pathways, restoring Treg/Th17 cell balance and the gut microbiota, thus prevented disease progression of colitis. Our findings demonstrate that edible nanovesicles (EVs) in dairy products such as milk have intrinsic immunomodulatory functions beneficial to the gut. However, from a practical point of view, to reach the therapeutic dose of mEVs (e.g. 600 µg/kg body weight per day for the treatment of colitis), a human adult may need to drink 1 liter of fresh milk per day. Therefore, it is conceivable that a diet formula supplemented with mEVs may be of great potential to protect and preserve gut's health.

## Supplementary Material

Supplementary figures and tables.

Supplementary files.
