# Supplementary material for: Milk-derived extracellular vesicles alleviate ulcerative colitis by regulating the gut immunity and reshaping the gut microbiota
Source: Theranostics. 2021 Jul 25;11(17):8570–86. doi: 10.7150/thno.62046 (PMC8344018; doi:10.7150/thno.62046)
Supplement: Supplementary file 1 — Supplementary figures and tables. [file thnov11p8570s1.pdf]

Milk-derived extracellular vesicles alleviate ulcerative colitis by regulating the gut immunity and reshaping the gut microbiota

Lingjun Tong[1,2,3], Haining Hao[1], Zhe Zhang[1], Youyou Lv[1], Xi Liang[1], Qiqi Liu[1], Tongjie Liu[1], Pimin Gong[1], Lanwei Zhang[1], Fangfang Cao[4], Giorgia Pastorin[5], Chuen Neng Lee[2,3], Xiaoyuan Chen[2,3,4,6,7], Jiong-Wei Wang[2,3,8,9*], Huaxi Yi[1,10*]

[1]College of Food Science and Engineering, Ocean University of China, 5 Yushan Road, Qingdao 266003, P. R. China

[2]Department of Surgery, Yong Loo Lin School of Medicine, National University of Singapore, 1E Kent Ridge Road, Singapore 119228, Singapore

[3]Nanomedicine Translational Research Programme, Centre for NanoMedicine, Yong Loo Lin School of Medicine, National University of Singapore, Singapore 117609, Singapore

[4]Departments of Diagnostic Radiology, Yong Loo Lin School of Medicine, National University of Singapore, Singapore 119074, Singapore

[5]Department of Pharmacy, Faculty of Science, National University of Singapore, Singapore 117543, Singapore

[6]Department of Chemical and Biomolecular Engineering, and Department of Biomedical Engineering, Faculty of Engineering, National University of Singapore, Singapore 117575, Singapore

[7]Clinical Imaging Research Centre, Centre for Translational Medicine, Yong Loo Lin School of Medicine, National University of Singapore, Singapore 117599, Singapore

[8]Cardiovascular Research Institute (CVRI), National University Heart Centre Singapore (NUHCS), 14 Medical Drive, Singapore 117599, Singapore

[9]Department of Physiology, Yong Loo Lin School of Medicine, National University of Singapore, 2 Medical Drive, Singapore 117593, Singapore

[10]Key Laboratory of Precision Nutrition and Food Quality, Department of Nutrition and Health, China Agricultural University, Beijing 100083, China

* Correspondence to: yihx@ouc.edu.cn (H.X.Y.) and surwang@nus.edu.sg (J.W.W.)
Tel.: +86-0532-8203-2162 (H.X. Y.); +65-6601-1387 (J.W.W.)

Supplementary material

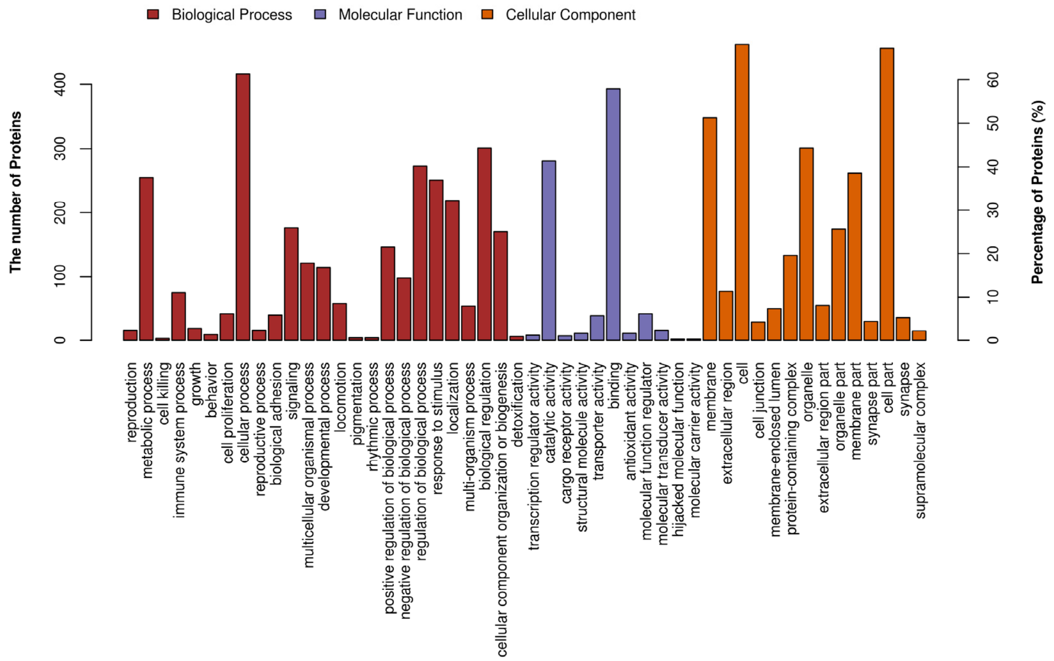

Figure S1. Gene Ontology analysis of the differentially expressed proteins in mEVs.

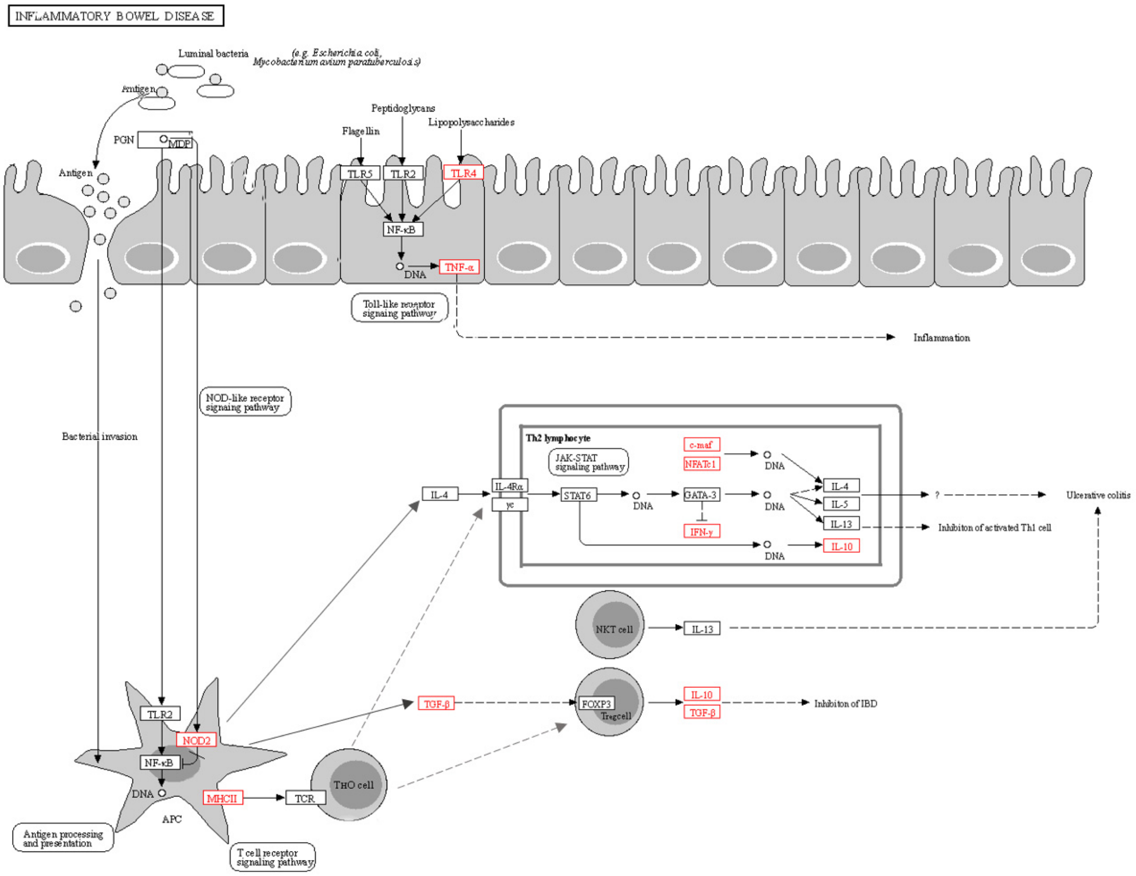

Figure S2. The signaling pathways in ulcerative colitis potentially targeted by mEV miRNAs.

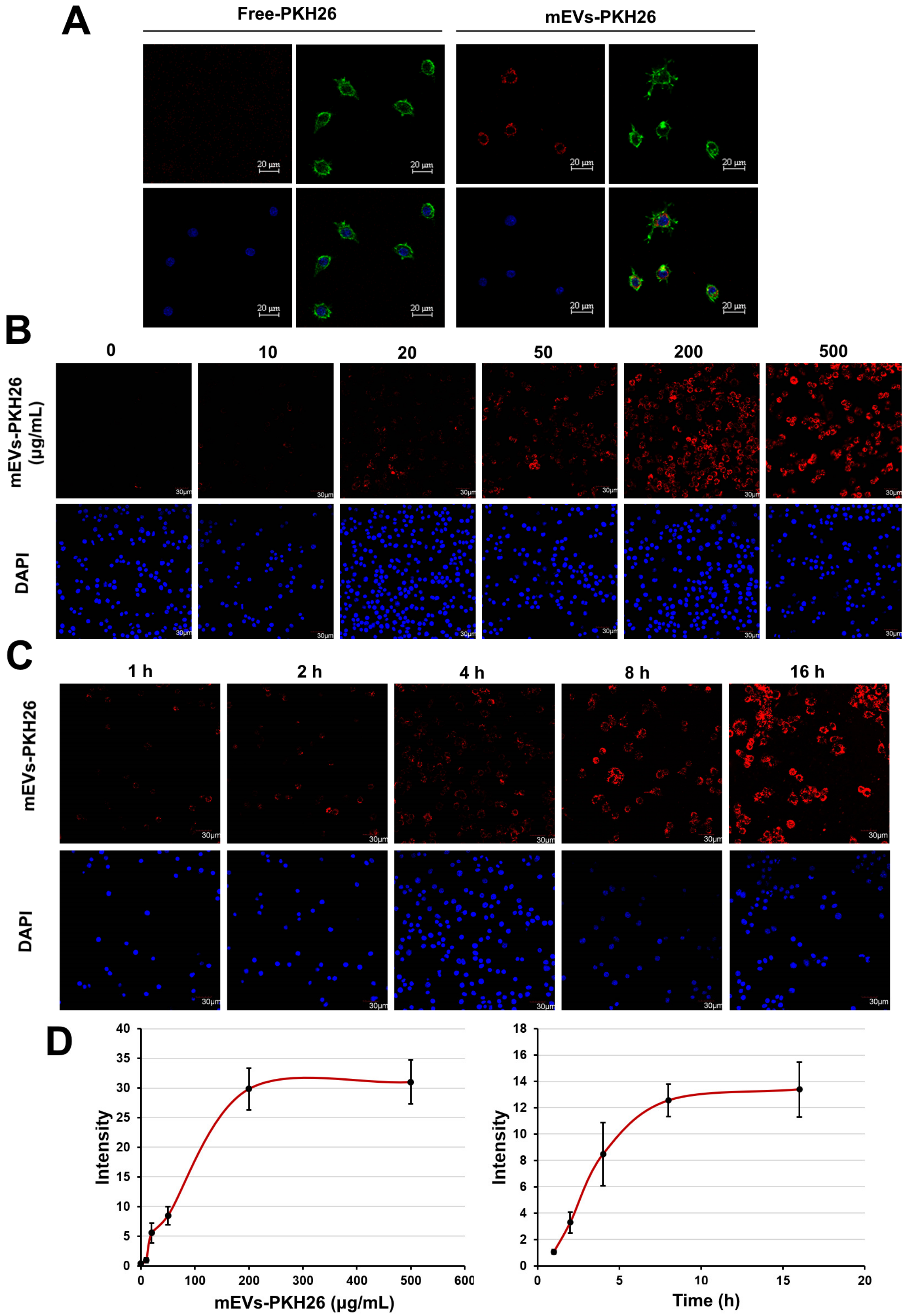

Figure S3. Uptake of mEVs by RAW264.7 cells. The nucleus was labeled with DAPI (blue), actin filaments were labeled with FITC Phalloidin (green), and mEVs were labeled with PKH26 (red). (A) PKH26-labeled mEVs (500 μg/mL) were added into confocal dishes (5×10^4 cells/dish) and incubated at 37 °C for 4 h; PBS-PKH26 (free

dye) was used as the control. (B) The effect of mEV dose (0 - 500 μg/mL) on uptake of mEVs. (C) The effect of incubation time (1, 2, 4, 8 and 16 h) on uptake of mEVs (200 μg/mL). (D) The dose and time-dependent curves of mEV uptake by RAW264.7 cells. The fluorescent intensity of PKH26 (mEV labeling) was quantified using Image J (n = 3).

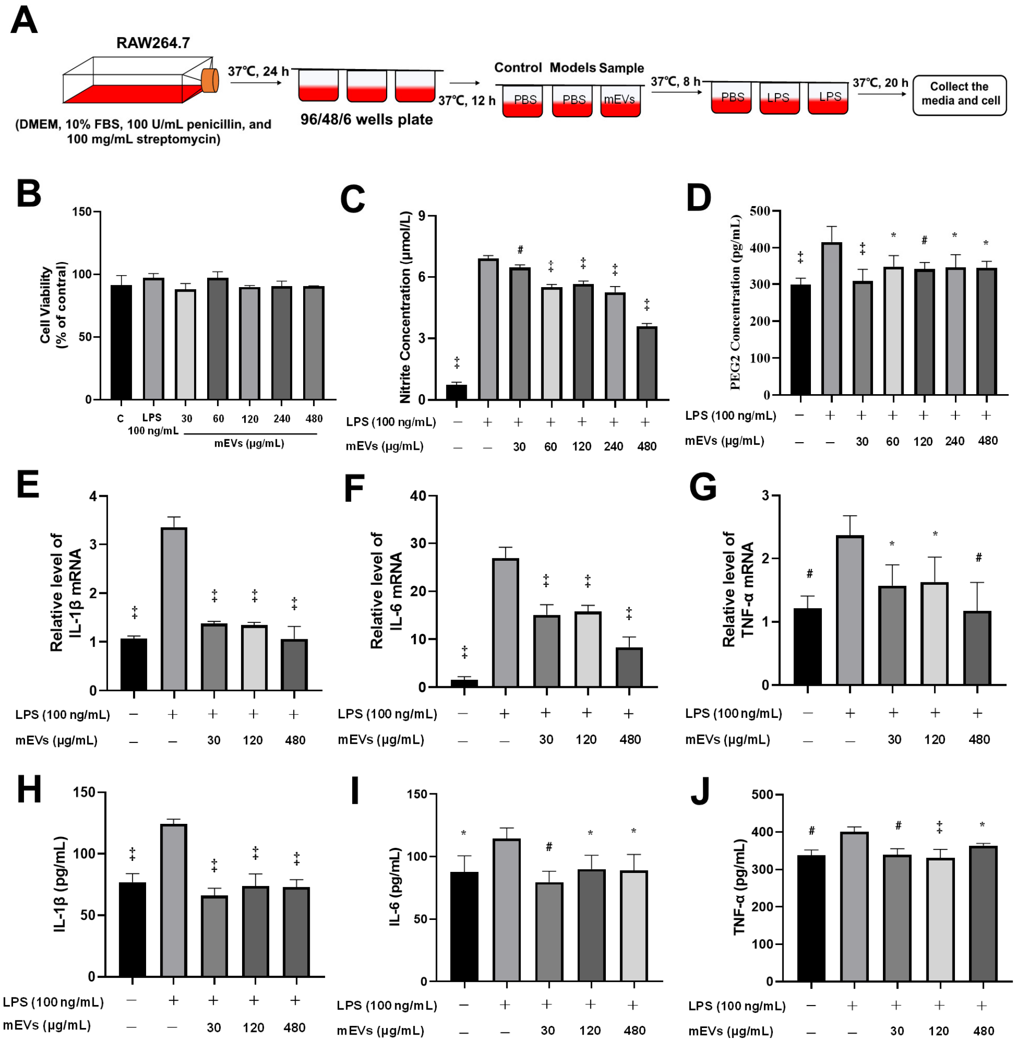

Figure S4. Immunomodulatory effects of mEVs *in vitro*. (A) A schematic diagram illustrating the experimental steps *in vitro*. (B) The cell viability assessed by MTT assay. (C, D) The effects of mEVs on the production of NO and PGE2. (E-G) mRNA expression of immune cytokines. GAPDH was used as the reference gene. (H-J) Secreted protein levels of cytokines IL-1β, IL-6 and TNF-α in the culture medium. Data were obtained from three independent experiments and presented as mean ± SD (n = 3). $^{*}p < 0.05$, $^{\#}p < 0.01$ and $^{‡}p < 0.001$ *vs.* LPS model group.

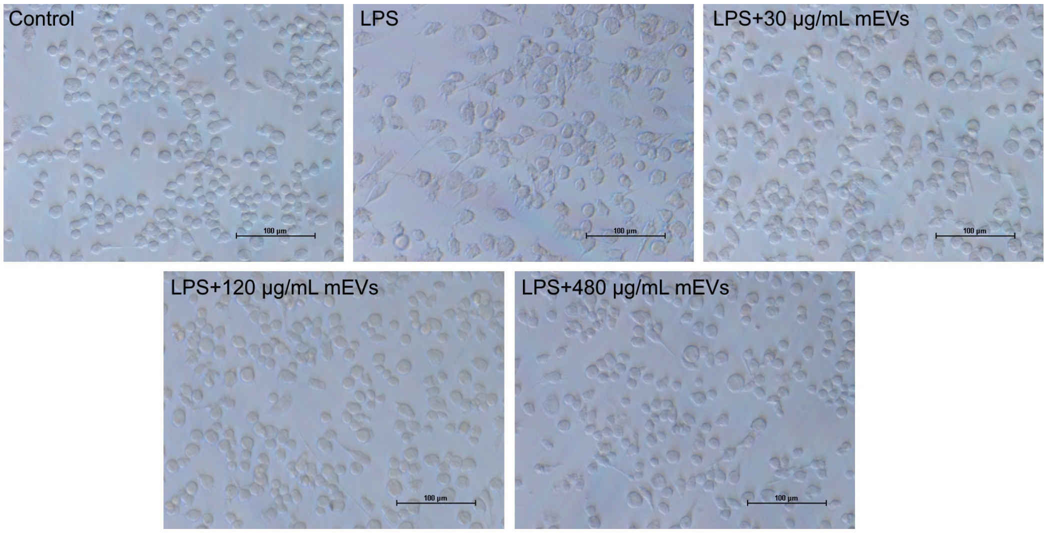

Figure S5. Representative images showing changes in the morphology of LPS-stimulated RAW264.7 cells. Cells were incubated in the presence or absence of mEVs for 8 h. Images were taken under fluorescent inverted microscope. Scale bars represent 100 μm.

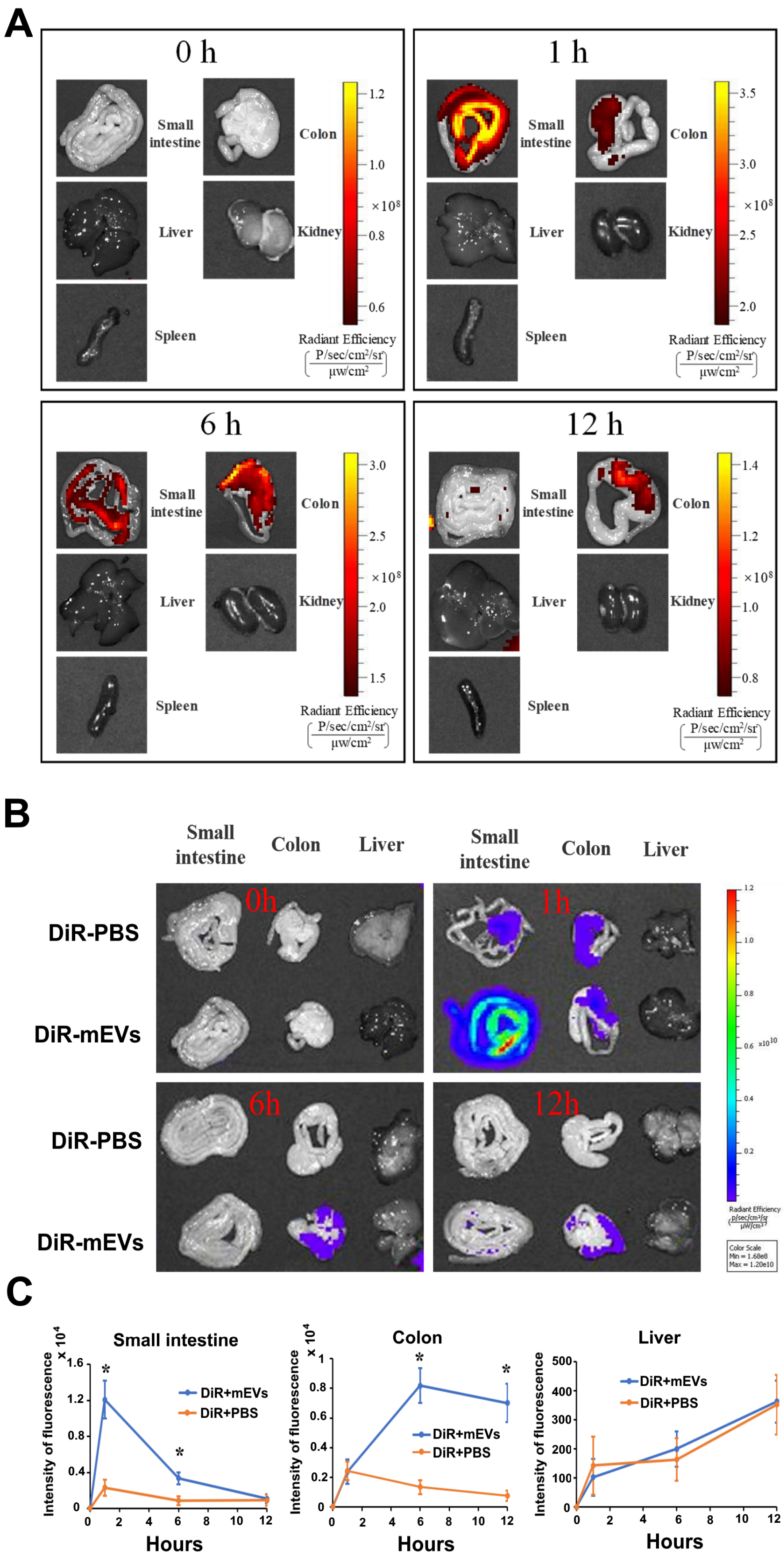
A
0 h
1 h
6 h
12 h
Small intestine
Colon
Liver
Kidney
Spleen
Radiant Efficiency
P/sec/cm²/sr / μw/cm²
×10⁸
B
Small intestine
Colon
Liver
DiR-PBS
DiR-mEVs
0h
1h
6h
12h
C
Small intestine
Colon
Liver
DiR+mEVs
DiR+PBS
Intensity of fluorescence
Hours

Figure S6. *In vivo* biodistribution of mEVs after oral administration. (A) Accumulation of mEVs in different organs at different time points. Mice were gavage-administered DiR–labeled mEVs (0.5 mg/mouse) and imaged over 12 h by IVIS imaging system. To visualize the various amounts of mEVs, different scale bars were used at different time points. (B) Accumulation of mEVs in different organs (the duodenum, colon and liver) at different time points following a gavage of DiR-labeled mEVs or DiR-labeled PBS (free dye control). The same fluorescence intensity scale bar was applied to all images for easy comparison. (C) Quantitative analysis of fluorescence intensity of mEVs accumulated in different organs using Image J software. N = 4 mice per time point per treatment group. $^{*}p < 0.05$ compared with free dye (DiR-PBS) group.

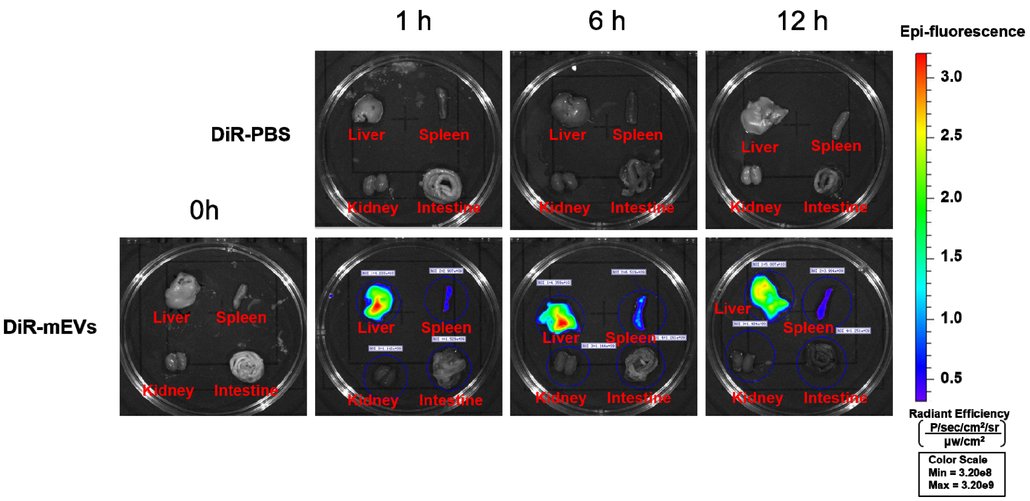

Figure S7. *In vivo* biodistribution of mEVs after intravenous administration. DiR-labelled mEVs (0.5 mg/mouse) or free dye in PBS (DiR-PBS) were injected through the tail vein. Accumulation of mEVs or free dye in different organs at different time points was analyzed by IVIS imaging system over 12 h.

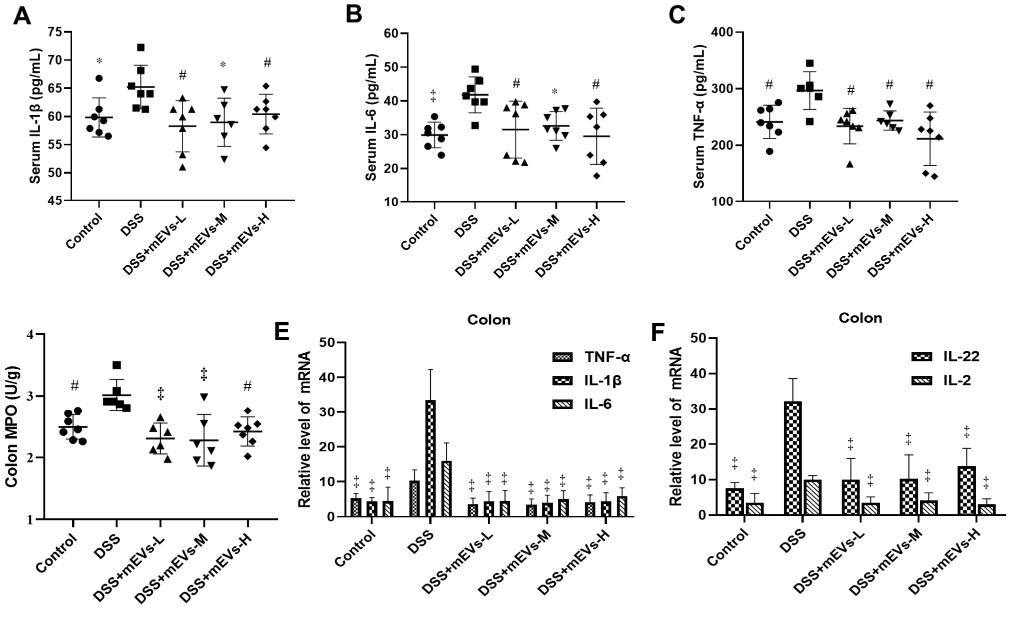

Figure S8. mEVs inhibit production of immune cytokines in DSS-induced colitis mice. (A-C) Serum levels of IL-1β, IL-6 and TNF-α measured by ELISA kits. Data were presented as mean ± SD (n = 7). (D) MPO activity in colon tissue determined by an ELISA kit. Data were presented as mean ± SD (n = 7). (E, F) Gene expression levels of IL-1β, IL-6, IL-2, IL-22 and TNF-α in the colon determined by qPCR. Data were presented as mean ± SD (n = 5). $^{*}p < 0.05$, $^{\#}p < 0.01$ and $^{‡}p < 0.001$ *vs.* DSS group.

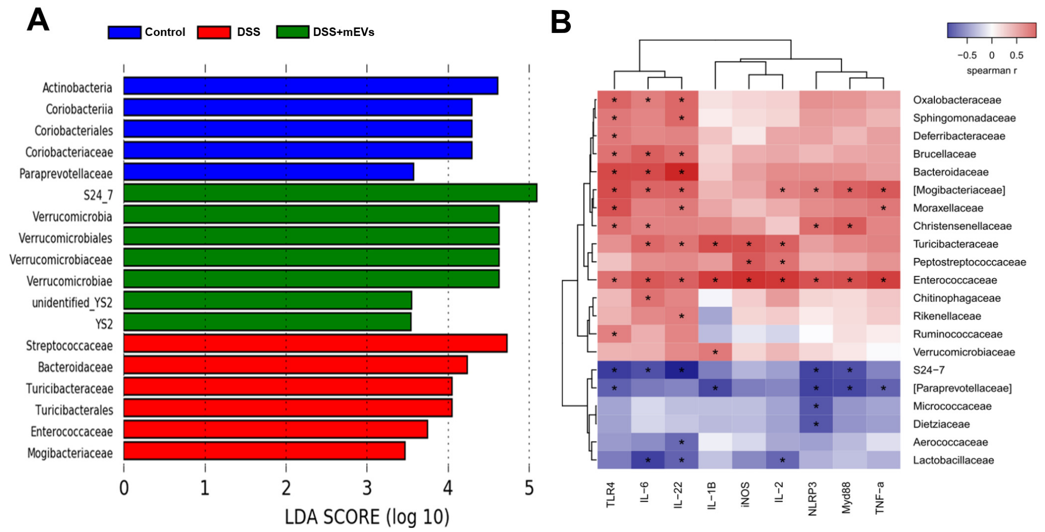

Figure S9. Correlation analysis of the gut microbiota and immune inflammatory factors. (A) Differentially enriched gut microbiota in each group of mice at the family level by linear discriminant analysis (LDA). (B) Correlation matrix showing the strength of correlation between the gut microbiota (at the family level)-immune inflammatory factors in the colon. Values in cells are Spearman correlation coefficient (Spearman r). Statistical significance was determined for all pairwise comparisons using Spearman's method. *$p < 0.05$. Spearman r values range from -0.5 (blue) to 0.5 (red).

Table S1. EV-associated proteins identified in mEVs.

Family	Protein name
Tetraspanins	CD63, CD81, CD82
MHC class I	MHC class I antigen, MHC class I heavy chain
Complement-binding proteins	CD59
EMMPRIN	BSG
ESCRT-I/II/III	TSG101, CHMP*
Rab proteins	Rab-25, RAB14, Rab18, etc.
Heat shock proteins	HSP90, HSP70
Annexin	Annexin A1, A4, A5, A7, etc.

* The Charged Multivesicular Body Protein (CHMP).

Table S2. Number of mEV proteins involved in the immune inflammation pathways.

Map_ID	Map Name	Number of proteins
bta04014	Ras signaling pathway	23
bta05163	Human cytomegalovirus infection	21
bta04151	PI3K-Akt signaling pathway	19
bta04062	Chemokine signaling pathway	19
bta04015	Rap1 signaling pathway	18
bta04145	Phagosome	17
bta04010	MAPK signaling pathway	15
bta04722	Neurotrophin signaling pathway	13
bta04024	cAMP signaling pathway	12
bta04152	AMPK signaling pathway	10
bta04621	NOD-like receptor signaling pathway	8
bta04130	SNARE interactions in vesicular transport	6
bta04660	T cell receptor signaling pathway	6
bta04662	B cell receptor signaling pathway	6
bta04630	JAK-STAT signaling pathway	5
bta04620	Toll-like receptor signaling pathway	5
bta04750	Inflammatory mediator regulation of TRP channels	4
bta04370	VEGF signaling pathway	4
bta04064	NF-kappa B signaling pathway	3
bta04657	IL-17 signaling pathway	3
bta04672	Intestinal immune network for IgA production	2
bta05321	Inflammatory bowel disease (IBD)	2
bta05320	Autoimmune thyroid disease	2
Total		223

Table S3. Number of mEV miRNAs (Top 100 miRNAs) targeting the immune inflammation pathways/inflammatory bowel disease (IBD).

Number	miRNA	Targeting inflammation pathway	Targeting IBD
1	miR-148a	√	√
2	miR-186	√	
3	miR-27b	√	√
4	miR-141	√	
5	miR-339b	√	
6	miR-125b	√	
7	miR-2285t	√	
8	miR-151-3p	√	
9	miR-423-5p	√	
10	miR-375	√	
11	miR-152	√	√
12	miR-10174-3p	√	√
13	miR-185	√	
14	miR-2478	√	
15	miR-660	√	
16	miR-429	√	
17	miR-182	√	√
18	miR-652	√	
19	miR-19b	√	
20	miR-1839	√	
21	let-7a-3p	√	
22	miR-106b	√	√
23	miR-194	√	
24	miR-2284x	√	√
25	miR-484	√	
26	miR-1260b	√	
27	miR-374b	√	
28	miR-342	√	
29	miR-28	√	√
30	miR-6524	√	
31	miR-22-5p	√	
32	miR-11986b	√	√
33	miR-2285bf	√	
34	bta-miR-143	√	
35	miR-7	√	
36	miR-142-5p	√	√

Table S4. Primer sequences for qRT-PCR analysis.

Gene	Forward Primer Sequence	Reverse Primer Sequence
TNF-α	GCGACGTGGAACTGGCAGAAG	GCCACAAGCAGGAATGAGAAGAGG
IL-1β	TCGCAGCAGCACATCAACAAGAG	TGCTCATGTCCTCATCCTGGAAGG
IL-2	GCAGCTCGCATCCTGTGTCAC	CTGCTGTGCTTCCGCTGTAGAG
IL-6	ACTTCCATCCAGTTGCCTTCTTGG	TTAAGCCTCCGACTTGTGAAGTGG
IL-22	TCCAACTTCCAGCAGCCATACATC	GCACTGATCCTTAGCACTGACTCC
IL-10	GAGGATCAGCAGGGGCCAGTAC	AAGGCAGTCCGCAGCTCTAGG
TLR-4	ACAAGGCATGGCATGGCTTACAC	TGTCTCCACAGCCACCAGATTCTC
MyD88	GCTAGAGCTGCTGGCCTTGTTAG	TCTCGGACTCCTGGTTCTGCTG
iNOS	TGCCACGGACGAGACGGATAG	CTCTTCAAGCACCTCCAGGAACG
NLRP3	GAGCTGGACCTCAGTGACAATGC	ACCAATGCGAGATCCTGACAACAC
GAPDH	GGTTGTCTCCTGCGACTTCA	TGGTCCAGGGTTTCTTACTCC

Table S5. The mouse dietary ingredients.

Numbers	Component
1	corn
2	soybean meal
3	flour
4	wheat middlings
5	fish meal
6	plant oil
7	dicalcium phosphate
8	limestone
9	salt
10	vitamins
11	mineral elements
